# Characterizing the Smell of Marijuana by Odor Impact of Volatile Compounds: An Application of Simultaneous Chemical and Sensory Analysis

**DOI:** 10.1371/journal.pone.0144160

**Published:** 2015-12-10

**Authors:** Somchai Rice, Jacek A. Koziel

**Affiliations:** 1 Interdepartmental Toxicology Graduate Program, Iowa State University, Ames, Iowa, United States of America; 2 Agricultural and Biosystems Engineering, Iowa State University, Ames, Iowa, United States of America; Barnard College, Columbia University, UNITED STATES

## Abstract

Recent US legislation permitting recreational use of marijuana in certain states brings the use of marijuana odor as probable cause for search and seizure to the forefront of forensic science, once again. This study showed the use of solid-phase microextraction with multidimensional gas chromatography—mass spectrometry and simultaneous human olfaction to characterize the total aroma of marijuana. The application of odor activity analysis offers an explanation as to why high volatile chemical concentration does not equate to most potent odor impact of a certain compound. This suggests that more attention should be focused on highly odorous compounds typically present in low concentrations, such as nonanal, decanol, o-cymene, benzaldehyde, which have more potent odor impact than previously reported marijuana headspace volatiles.

## Introduction

Americans know the Fourth Amendment of the U.S. Constitution protects citizens from unreasonable search and seizure, without a warrant, by government bodies. Landmark legal cases have set a precedent of what is deemed probable cause (S1 Table). Courts are challenged to be consistent with using odor of marijuana as probable cause when recreational use is now legal in some states and illegal at the federal level. Previous research has been conducted, identifying the volatile organic compounds (VOC) present in the headspace of marijuana. The major components of total VOC in headspace of the plant material has been reported to consist of limonene [1–5], α-pinene [1, 3, 4, 6], β-pinene [1, 3, 4, 6], β-myrcene [1, 3–5], β-ocimene [2, 4], β-caryophyllene [2, 4–6], α-caryophyllene [4, 6], α-phellandrene [4], 3-carene [4], α–terpinene [4], terpinolene [4], terpineol [5], linalool [4, 5], α-cadinene [4]. With improved analytical techniques, the list of identified compounds is increasing, starting from 20 compounds in 1973 [1] with an addition of 10 new compounds since [1–6]. Even though more compounds have been identified, it has not increased understanding of forensic *odor*. To date, a total of approximately 31 compounds are known to be emitted from marijuana [1–6].

Solid phase microextraction (SPME) was used as a non-destructive, non-invasive, sampling device to collect volatiles permeated through packaging and responsible for ‘characteristic’ aroma of marijuana. The use of micro-sampling techniques in forensic science has been reviewed in Kabir (2013) [7]. SPME is favored due to a smaller requirement on sample size, eliminated use of organic solvents, portability, and lends itself to automation [7]. SPME is also best at reducing matrix effects inherent in forensic work with blood, plasma, and urine [8]. Headspace (HS) sampling using SPME for characterization of volatile organic compounds (VOC) has been used to characterize explosives [9], confiscated 3, 4-methylenedioxy-N-methylamphetamine (MDMA a.k.a. Ecstasy), amphetamine [10], and cocaine [11]. The upsurge in the use of SPME as an all-in-one sample preparation, cleanup, and pre-concentration of volatiles in forensics highlights its importance to the field.

There are some clear favorites in instrumentation being used for analysis of headspace VOC emitted from marijuana. Gas-chromatography (GC) was used to try and distinguish marijuana of different geographic origins, with unsuccessful results for classification [12]. GC tandem mass-spectrometry (MS) was used to characterize volatile oil composition of dried and fresh marijuana buds [13], and to discern differences between volatile compounds found in male and female marijuana plants of Northern Lights and Hawaiian Indica [14]. Volatile composition of entire inflorescences of hemp have been analyzed by GC-MS [15], even with ultrasound-assisted extraction [4].

Dogs trained for specific odor detection (e.g. narcotics, explosives, cadavers) are the current benchmark used in the law enforcement community [16–18]. A study by Macias, *et al*. in 2008 [17] showed that a mixture of α-pinene, β-pinene, myrcene, limonene, and β-caryophyllene associated with marijuana showed low alert responses when field tested on narcotic detection dogs. None of the dogs alerted to Sigma Pseudo Marijuana scent [17] (Sigma Aldrich, St. Louis, MO, USA). In a separate study by Jezierski (2014) [18] comparing dogs trained and tested with illicit drugs, (i.e., 68 Labrador retrievers, 61 German shepherds, 25 terriers and 10 English cocker spaniels), it was found that German shepherds were superior scent dogs and terriers were inferior at detecting drugs. The researchers tested 5 types of illicit drugs and found that marijuana was the easiest for all dogs to detect, followed by hashish, amphetamine, cocaine, and lastly heroin. In over 1000 trials, the dogs found the hidden drugs within 64 sec and an 87.7% accuracy rate (5.3% false positive) [17]. It has also been shown that the dog handler may also affect alert responses, with a failure rate of 85% false positives during search of a clean room [19]. With such a large range of variability, research is warranted on discovering what triggers an alert from the dogs. Rice (2015) [20] and Rice and Koziel (2015) [21] have reported on the usefulness of using OAV to characterize forensic odor from drugs, and offer an explanation on why current surrogate scent training tools may not be effective for canines [21].

Is human sense of smell any better? In a situational based study by Dotty in 2004, subjects were asked to smell a garbage bag containing 5 pounds of marijuana, and a garbage bag of crushed newspapers [22]. All human subjects could identify the bag containing marijuana. Could these same people detect marijuana smell sitting in the driver’s compartment, with the marijuana in a garbage bag inside the car trunk? False positives (9.36%) was the same as true positives (12.97%), with p > 0.20, meaning there were no significant difference in detecting the marijuana bag versus the newspaper bag. Next, the researchers wanted to know if budding and non-budding marijuana plants produce similar odors (i.e. mature versus non-mature plants, respectively)? A tomato plant was used as the negative control. All participants found mature (budding) plant volatiles more intense (p< 0.025) suggesting the buds hold the odorous compounds. Intensities of immature cannabis did not differ significantly from the tomato plant. Lastly, the researchers wanted to test if the smell of marijuana can be distinguished when it is mixed with diesel exhaust. The rates of detection when combined with diesel exhaust were not significant [22].

Limited work has been published on canine and human detection of marijuana odor, yielding mixed results and high variability. A thorough, analytical approach to the investigation of marijuana odor detected by humans is warranted, if and when more states seek to legalize recreational use. The objectives of this study were to (1) identify odorous compounds emitted from marijuana using multidimensional gas chromatography (MDGC) tandem mass spectrometry coupled with simultaneous *human olfaction* and (2) show an application and novelty of odor activity values (OAV) to better understand the ‘characteristic’ aromas of marijuana (3) explore aromatic compounds that are emitted through packaging typical in illicit distribution of marijuana. The working hypothesis is that simultaneous chemical and sensory analysis can indicate the identity of aromatic compounds that are responsible for the characteristic smell of marijuana. This information is needed to (a) better understand which compounds are really responsible for the “characteristic” aroma of marijuana, (b) provide additional insight into aroma perception by applying a method (i.e., OAV) established in food and beverage field in a new setting (i.e., forensic sciences), and (c) investigate how marijuana packaged for illicit distribution can smell differently according to these OAV.

### Odor activity value

Odor perception is multi-faceted and this laboratory has highlighted this complexity, showing the role of highly odorous compounds present at extremely low concentrations [23]. There are two big hurdles when using GC for characterization of odorous compounds, sufficient resolution between aromatic compounds, and co-elution of two or more of these compounds. A GC using a non-polar column connected in series to a polar analytical column can account for such occurrences [24, 25]. The use of state-of-the-art *simultaneous* MDGC-MS-O allows researchers to separate, at high resolution, odors that may not be separated on a single column, and to detect compounds [24] based on their OAV. This report is the first instance of using MDGC-MS-O to characterize the odor of marijuana.

Since the introduction of GC—olfactometry (GC-O), intensity and odor character of an individual compound has been better described [26]. Patton and Josephson originally presented the concept of the OAV [27]. A caveat is offered for equating high chemical concentration to high odor impact. The quantitative measurements of chemical concentration have been the primary data collected to date, while qualitative measurements of odor character has been largely ignored in analysis of marijuana odor.

OAV=[Concentration]/ODT (1)

where ODT is odor detection threshold and defined as the concentration a compound is detected by 50% of the population [28]

OAV has been used extensively in the food and beverage industry to characterize aroma of bread, beef, coffee, beer [29] and wines [30, 31] and more recently odor emissions from animal buildings [32]. This report is the first application of OAV to characterize marijuana. This paradigm shift from concentration based (i.e., high concentration equates to potent odor) to OAV based aroma detection of marijuana and associated odor perception can help extend the knowledge of marijuana odor and its role in forensic science.

## Materials and Methods

The marijuana samples were obtained from Iowa Division of Criminal Investigation (Iowa DCI), Drug Identification Section. Marijuana was available in various states of seizure and included: 1) a US military-style duffel bag filled with marijuana weighing ~ 50 kg; 2) 1 gram air-dried marijuana (*loose*); 3) 1 gram of the same air-dried marijuana placed in a plastic zip-top sandwich bag (*bagged*).

Carboxen/Polydimethylsiloxane (PDMS), 85 μm Stableflex, 24 gauge solid-phase microextraction (SPME) fibers were used (Sigma-Aldrich, St. Louis, MO, USA). Briefly, experimental conditions were as follows: the drugs were placed in separate, pre-cleaned and baked 16 ounce (473 mL) mason jars with modified lids. The Carboxen/PDMS fibers were exposed to the headspace and volatiles were collected. Marijuana samples were placed in the sample jars and sealed, with the exception of the marijuana in a duffel bag (S1 and S2 Figs). Immediately, SPME fibers were inserted into the sample port and exposed for 5 min, 1 h, and 68 h at ambient temperature. When the extraction step was completed, the SPME fiber was retracted, wrapped in pre-baked aluminum foil, placed in a pre-cleaned mason jar, and transported back to the laboratory in a cooler on ice. In the laboratory, fibers loaded with VOC were stored in a 4°C refrigerator until analysis, wrapped in the foil and sealed in a clean mason jar. SPME fibers were exposed to the heated injection port of the MDGC-MS-O for thermal desorption and analysis.

MDGC-MS-O analysis was performed on an Agilent 6890 GC, with a restrictor guard column, non-polar capillary column (BP-5, 56 m x 530 μm inner diameter x 1.00 μm thickness, SGE, Austin, TX, USA) and polar capillary column (BP-20, 25 m x 530 μm inner diameter x 1.00 μm thickness, SGE, Austin, TX, USA) connected in series. Outflow from analytical column was held at 7.0 mL/min. Sample flow was split 3:1 via open split interface to the sniff port and mass spectrometer, respectively, as determined by restrictor column inner diameter. Desorption time was 2 min in splitless mode at 270°C under flow of helium carrier gas (99.995% purity). Subsequent analysis of the same fiber immediately afterward, revealed no carry over. The oven temperature was programmed as follows: 40°C for 3.00 min, then increased to 220°C at a rate of 7.00°C per min, and held for 11.29 min (40 min total run time). The carrier gas was set at constant pressure at the midpoint (junction point of the non-polar and polar column) at 5.8 psi (0.395 atm). Restricted transfer line to the MS was set at 240°C; restricted transfer line to the sniff port was set at 240°C with humidified air flow. MS heated zones were 150°C for the quadrupole and 230°C for the source. The MS source was electron ionization mode with ionization energy set at 70 eV. Mass acquisition range m/z 33.0–280.0 u.

Tentative identification of VOC was performed using the Automatic Mass Spectral Deconvolution and Identification System (AMDIS) (National Institute of Standards and Technology, Gaithersburg, MD) and six specialty mass spectral libraries provided derived from the NIST05/EPA/NIH mass spectral database. It was not appropriate to use retention indexes (Kovats RI) for identification due to the configuration of the capillary columns, but known retention times of standards previously analyzed on this system were also used for identification.

One panelist was trained using odorous chemical standards on MDGCMS-O. Sample availability and time allotted at the crime lab allowed for limited fibers (i.e., limited replicates). A single panelist analyzed all sample fibers in the open experiments. A maximum of 6 sample fibers was analyzed per day. There were three parameters recorded for perception of odorants during olfactometry work outlined in this study. The first parameter was detectability, defined here as the minimum concentration of the odorant needed to be recognized. Secondly, intensity for each aroma note was recorded, and defined here as the perceived strength of the aroma event. Guidelines for a unitless, relative intensity scale were as follows: not present = 0, faint = 25, distinct = 50, strong = 75, intense = 100. The last parameter measured was character, or aroma descriptor, as described by the trained panelist. A descriptor of “characteristic” was used when an odor was distinguished to represent the overall aroma of the sample. Area under the peak of each aroma event in the aromagram is calculated as Aroma Area = Width x Intensity x 100, where width is the length of time in min that an aroma persisted.

## Results

The instrument was tuned daily and column blanks were performed and did not show any contaminating compounds. Final analysis of blank trip fibers (an unloaded SPME fiber taken to the site and back, exposed to a clean jar with no sample, and stored with sampled fibers to be analyzed) did not show any contaminating compounds. Deconvolution resolution in AMDIS was medium, and signal to noise ratio ranged from 5 to 937 (not shown). In this study, a total of 233 compounds were tentatively identified as volatiles in headspace emitted from marijuana at room temperature (Table 1). This list was compiled from analysis of lab-stored, desiccated marijuana (S1 Fig) in packaged and unpackaged form, and newly seized, fresh marijuana (S2 Fig). Over 200 new compounds were added to the list of volatiles known to be emitted from marijuana. Newly reported compounds, represent an addition of 95% of the total compounds reported in Table 1. In this research, the authors are not differentiating between VOC emitted from marijuana samples and VOC emitted from packaging. Due to the legal nature of the samples from active cases, the authors were not able to disturb the evidenced samples or obtain true “blank” plastic bags or duffel bags. Also, from the context of total forensic odor from OAV and not concentration in HS, differentiating between such VOC may not be not be useful if the differences in odor impact of the compounds are small. Full details including CAS number, retention times, significant ions, % spectral match, aroma descriptors, published ODT, relative abundance (given as peak area counts), and calculated OAV for each compound are given in S2 and S3 Tables.

**Table 1:** Comparison of (a) 233 volatiles found in this study emitted from marijuana, including those emitted through-packaging with (b) volatiles previously reported as ‘signature’ compounds of marijuana in headspace.

	(-)-Aristolene	(-)-Globulol	(+)-4-Carene	(+)-calarene	(+)-nerolidol
	(+)-sativene	(1R)-(+)-trans-isolimonene	1-(3-methylphenyl)-ethanone	1-(3-methylphenyl)-ethanone	1,1-dimethyl-hydrazine
	1,2,3,4-tetramethylbenzene	1,2-diethylbenzene	1,3,5-triazine-2,4,6-triamine	1,3-dichlorobenzene	1,4-diethylbenzene
	1-butanol	1-butoxy-2-propanol	1-hexadecanol	1-hexanol	1-undecanol
	2,2,5-trimethylhexane	2,3,4-trimethylpentane	2,4,6-trimethylphenol	2,4-di-tert-butylphenol	2,6-diethylpyrazine
	2,6-dimethylquinoline	2-butanone	2-butoxyethanol	2-chloroacetophenone	2-ethenyl-1,3-dimethylbenzene
	2-ethoxyethanol	2-ethylhexanol	2-ethyltoluene	2-heptanone	2-hydroxyacetophenone
	2-isopropenyl-3-methylpyrazine	2-methyl naphthalene	2-methyl-1H-imidazole	2-methyl-2-propanamine	2-methylaziridine
	2-methylpentane	2-nitropropane	2-phenoxyethanol	3,4,5-trimethyl-1-hexene	3,4,5-trimethylphenol
	3-ethyl-o-xylene	3-ethyltoluene	3-isopropylbenzaldehyde	3-methyl-2-cyclopenten-1-one	3-methylheptane
	3-methylpentane	3-pentanol	4-ethoxy-3-anisaldehyde	4-methyl guaiacol	4-methyldecane
	4-methylphenethylamine	4-methylpyrimidine	4-pyridinamine	5-ethenyl-2-methylpyridine	5-methylindane
	5-octanolide	7-methoxycoumarin	Acetaldehyde	Acetamide	Acetic acid
	Acetone	Acetophenone	Acrolein	Alloaromadendrene	Anethole
	Aromadendrene	Benzaldehyde	Benzonitrile	Benzophenone	Benzphentamine
	Benzyl acetate	Benzyl Alcohol	Benzyl formate	Benzyl nitrile	Betahistine
**This Study (a)**	Betazole	Butane	Butyl formate	**Camphene•**	Carbofuran
	Carvacrol	**Caryophyllene oxide•**	Cedryl acetate	cis-2-pinanol	Citronellolformate
	Citronellyl acetate	Cumene	Cuminaldehyde	Decanal	Diacetone alcohol
	Dibutyl phthalate	Diethyl Phthalate	Dimethylbenzylcarbinyl acetate	Dimethylpyrazine	Dimethylsulfide
	Dimethylsulfone	DL-carvone	Dodecane	Durene	Dyclocaine
	Estragole	Ethanol	Ethylacetate	Ethylene oxide	Ethylenediamine
	Ethylenimine	Eugenol	Eugenyl acetate	**Fenchyl alcohol•**	Formic acid
	Furfural	Furfurylmethylamphetamine	Heptanal	Hexadecane	Hexanal
	Hexanoic acid, methyl ester	Hexanoic acid, propyl ester	Hexestrol	Hordenine	Hydrazine
	Isoamyl alcohol	Isobornyl acetate	Isobornyl thiocyanoacetate	Isobutane	Isobutyraldehyde
	Isobutyrophenone	Isocyanatomethane	Isodurene	Isoeugenol	Isoprene
	Isoquinoline	**Limonene•**	Limonene dioxide	**Linalool•**	Linalyl acetate
	Longifolene	m-cymene	Methacrolein	Methacrylic anhydride	Methyl acetate
	Methyl acetylsalicylate	Methyl anthranilate	Methyl benzoate	Methyl heptadienone	Methyl heptanoate
	Methyl isoeugenol	Methyl mercaptan	Methyl salicylate	Methyl valerate	Methylene chloride
	methylhydrazine	Methylisohexenyl ketone	m-tert-butylphenol	Myrcene	Nerol
	Nerolidol	Nitrobenzene	Nonanal	Nonane	Octanal
	o-cymene	o-dimethyl hydroquinone	o-guaiacol	o-methylacetophenone	o-xylene
	p-acetanisole	p-aminotoluene	**p-cymene•**	Pentadecane	Pentamethylbenzene
	Pentanal	Perillaldehyde	p-ethyltoluene	Phenol	Phenylethyl alcohol
	Piperidine	Piperonal	p-methylacetophenone	Propanal	Propanoic acid, anhydride
	Propofol	Propylamine	Propylene glycol	p-tert-butylphenol	p-xylene
	Sabinene	Salicyladehyde	Styrene	**Terpinolene•**	tert-butanol
	tert-butyl-benzene	Tetrahydrozoline	Thymol	Toluene	Tridecane
	Tyramine	Undecane	**Valencene•**	Verbenone	α-bisabolol
	α-bulnescene	**α-cadinene•**	α-cedrene	α-copaene	α-cubebene
	α-guaiene	α-gurjunene	**α-humulene•**	α-ionol	α-longipinene
	α-methylcinnamaldehyde	**α-phellandrene•**	**α-pinene•**	**α-terpinene•**	α-terpineol
	**β-caryophyllene•**	β-cedrene	β-irone	**β-pinene•**	**β-selinene•**
	γ-gurjunene	γ-hexalactone	**γ-terpinene•**	**δ-3-carene•**	δ-cadinene
	3-(1-methylethyl)-phenol methylcarbamate	3-(3-hydroxyphenyl)-2-propenoic acid, methyl ester	3-methyl-5-(1-methylethyl)-Phenol methylcarbamate	1-Propanamine,3-dibenzo[b,e]thiepin-11(6H)-ylidene-N,N-dimethyl-, S-oxide	
**Previously reported (b)**					
	(E)-ocimene⊥	**Limonene•**	**Linalool•**	**Terpinolene•**	**α-cadinene•**
**Da Porto (2014) [4]**	**α-humulene•**	**α-phellandrene•**	**α-pinene•**	**α-terpinene•**	**β-caryophyllene•**
	β-myrcene⊥	**β-pinene•**	**δ-3-carene•**		
	1,8-Cineole⊥	3-Hexen-1-ol-acetate⊥	**Camphene•**	Cis-Hex-3-en-1-ol⊥	Eudesma-3,7(11)- diene⊥
**Rather (2011) [6]**	Guaiol	**Limonene•**	**Valencene•**	**α-humulene•**	**α-pinene•**
	**β-caryophyllene•**	β-chamigrene⊥	β-maaliene⊥	β-ocimene⊥	**β-pinene•**
	**β-selinene•**				
**Lai (2008) [2–3]**	**Limonene•**	**α-pinene•**	**β-caryophyllene•**	β-myrcene⊥	β-ocimene⊥
	**β-pinene•**				
**Osman (1985) [5]**	**β-caryophyllene•**				
	**Camphene•**	**Caryophyllene oxide•**	**Fenchyl alcohol•**	**Limonene•**	**Linalool•**
**Hood (1973) [1]**	Methyl heptenone⊥	**p-cymene•**	**Terpinolene•**	α-Bergamotene⊥	**α-humulene•**
	**α-pinene•**	**α-terpinene•**	**β-caryophyllene•**	β-Farnesene⊥	β-myrcene⊥
	β-ocimene⊥	β-phellandrene⊥	**β-pinene•**	**γ-terpinene•**	**δ-3-carene•**

**Bolded•** compounds indicate concurrent identification with this study and previously reported studies. Underlined⊥ compounds indicate compounds previously reported but not found in this study. No true “blank” package sample type was available for comparison of the same material and manufacturing lot of the seized illicit marijuana. Therefore, this report is conveying all compounds found in headspace of marijuana, regardless of packaging type and presence.

### Permeation of marijuana volatiles through packaging

**Fig 1:**
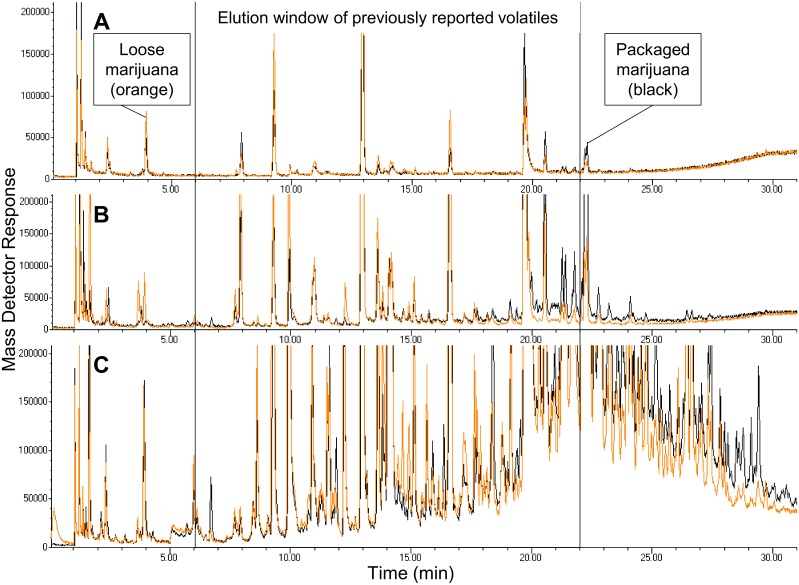
Effects of sampling time, as total ion chromatograms (TICs), of volatiles emitted from marijuana. Orange signal represents volatiles emitted from 1 g desiccated marijuana, loose in a 473 mL glass jar. Black signal represents volatiles emitted from 1 g desiccated marijuana, in a plastic zip-top bag, in a 473 mL glass jar. The boxed retention time widow highlights where volatiles, from previously published articles, would elute from the analytical column of the MDGC-MS-O. The set of 3 TICs represent volatiles extracted over (A) 5 min, (B) 1 h, and (C) 68 h resulting 20, 54, and 101 chromatographic peaks (orange signal), and 25, 39, 108 chromatographic peaks (black signal), respectively. See S2 and S3 Tables for the complete summary of identified compounds.

Lai, et al. (2008) reported 1–2 min sampling times were enough for detection of selected volatile markers in marijuana and cocaine. The target VOC were limonene and α/β- pinene, and β-myrcene [2]. The purpose of selecting short sampling time (i.e., 5 min) and long sampling time (i.e., 68 h) was to capture the full aroma profile of marijuana while taking into consideration the limitations of the Carboxen/PDMS SPME coating. Short sampling time allows for the adsorption of lower molecular weight (MW) VOC. Longer sampling time allows for adsorption of higher MW VOC, at the expense of lowered selectivity for the more volatile, lower MW VOC. Exploration of the effects of packaging and dwell time of marijuana in packaging (i.e. sampling time and storage/equilibrium time in the package were identical) revealed an increase in the number of chromatographic peaks detected, with increased headspace sampling time, in both the loose and bagged marijuana. Fig 1 shows an overlay of the total ion chromatogram (TIC) generated by the MS showcasing detected VOC emitted from in loose marijuana in a sealed glass jar and detected VOC emitted *through* a plastic zip-top sandwich bag in a sealed glass jar.

Across all 3 sampling times, 134 total volatiles were identified from headspace emitted from marijuana, through a plastic zip-top sandwich bag (S2 Table) and loose (S3 Table) with a net match of 65% or higher from AMDIS. Data analysis using all 6 specialty libraries provided in AMDIS resulted in 20, 54, and 101 chromatographic peaks identified in the loose marijuana and 25, 39 and 108 chromatographic peaks identified in the bagged for sampling times of 5 min, 1 h, and 68 h, respectively (Fig 1A–1C). Previously reported volatiles (bolded in Table 1) are known to elute between 6 min and 22 min on the MDGC-MS-O system used in this study (boxed in Fig 1). Please see S2 and S3 Tables for full details regarding the identification, odor character, and OAV of these 134 compounds. Results indicate that the number of unique VOC emitted into HS of marijuana stored at room temperature increased with time, whether packaged in a plastic sandwich bag or loose.

There was not a statistically significant effect of packaging on surrogate concentration of the 134 total VOC emitted from marijuana (p > 0.05). Surrogate concentration is defined as MS response to each separated compound, in peak area counts (PAC). There was statistical significant effect of sampling time on surrogate concentration for 34% of the VOC emitted from marijuana (p < 0.05). See S4 Table for full summary of F-statistics and p-values from statistical analysis. Compounds previously reported as key components of marijuana odor (α-humulene [4, 6] and β- caryophyllene [2, 4–6] did not permeate through packaging after 5 min. β- Caryophyllene [2, 4–6] did not permeate through packaging after 1 h. After 68 h of storage, 51 of 53 total compounds permeated through plastic packaging. Preliminary results show that packaging of marijuana in plastic zip-top sandwich bags does not have a significant effect on VOC emitted (i.e. odorous VOC), but storage time has a significant effect on the surrogate concentration of VOC emitted (i.e. more time allowed for more odorous VOC to be emitted). This suggests that diffusion of 134 volatiles through packaging was random (p > 0.05) but the amount (i.e. surrogate concentration) of these volatiles was affected by time. Specifically, by 68 h, the surrogate concentrations of volatiles emitted were significantly higher than 5 min, regardless of packaging. Marijuana recently stored in a plastic sandwich bag (i.e. 5 min) may have a different odor profile than marijuana stored in a plastic bag for 68 h, due to concentration differences that impact OAV.

### Application of OAV to marijuana volatiles

There were 124 chemical peaks tentatively identified using MDGC-MS, thought to be compounds emitted from marijuana through plastic zip-top sandwich bag regardless of sampling time (S2 Table). Only 8%, 19%, and 58% of the total compounds detected by MS after 5 min, 1 h, 68 h sampling time had published odor descriptors. Eight %, 11%, and 41% of the total compounds detected by MS after 5 min, 1 h, 68 h sampling time had published ODT. Similarly, a total of 121 chemical peaks were tentatively identified by MDGC-MS directly emitted from marijuana (a.k.a., loose) regardless of sampling time (S3 Table). Only 9%, 31%, and 59% of the total compounds detected by MS after 5 min, 1 h, 68 h sampling time had published odor descriptors. Seven %, 20% and 38% of the total compounds detected by MS after 5 min, 1 h, 68 h sampling time had published ODT. These numbers point out researchers only know as much as 59% of the information in terms of odor description, and 41% of the information in terms of ODT. Further research to reveal this missing information is warranted.

A Wilcoxon signed rank test of paired samples was performed (S5 Table) for each combination of time and packaging. This test compared the number of times when surrogate concentration is greater than calculated OAV, to the number of times when calculated OAV is greater than surrogate concentration, taking into account the size difference within the pairs. The null hypothesis is there is no difference in number of oppositions in each direction. Results indicated there is a significant difference between surrogate concentration and calculated OAV (using (Eq 1) and listed in S2 and S3 Tables) for loose marijuana at 1 h and 68 h extraction (p = 0.014 and p < 0.0001, respectively) and marijuana in a plastic zip-top bag at 68 h extraction (p < 0.0001). VOC were ranked by surrogate concentration (smallest surrogate concentration = 1) for bagged marijuana, shown in S6 Table. This illustrates how high chemical abundance does not equate to high odor intensity as perceived by human nose. Most importantly, compounds that have previously been reported as important volatile markers of marijuana based on high concentration and found in this study actually rank lower when using OAV (S3–S5 Figs) and vice versa. In other words, surrogate concentration of VOC and calculated OAV are not highly correlated (R2 < 0.638; See S7 Table). A general trend, based on available published human ODT, is that lower concentrated compounds could have more impact on odor, and therefore should be more responsible for the overall characteristic odor than the most concentrated compounds.

### Simultaneous chemical and sensory analysis of fresh marijuana

There were 179 compounds identified by MDGC-MS using AMDIS and 53 odor events associated with simultaneous olfactometry during a 68 h extraction of volatiles emitted from fresh marijuana through a cloth duffel bag (S2 Fig). Only 29% of the chemicals present in headspace of this marijuana sample registered an odor response by human nose. Only 31% of the total 179 compounds had published ODT in order to calculate OAV. Using Flavornet [33] and The Good Scents Company (TGSC) [34] aroma databases, 62% of the 179 compounds had a description of aroma perceived by human nose. This data highlights how almost 70% of the data presented in this study is missing crucial information (i.e. published ODT) needed to calculate OAV (1). See S8 Table for full details of all 179 compounds and 53 aroma events, associated aromas, ODT and calculated OAV for volatiles emitted from fresh evidence marijuana and emitted through a cloth duffel bag over 68 h.

**Fig 2:**
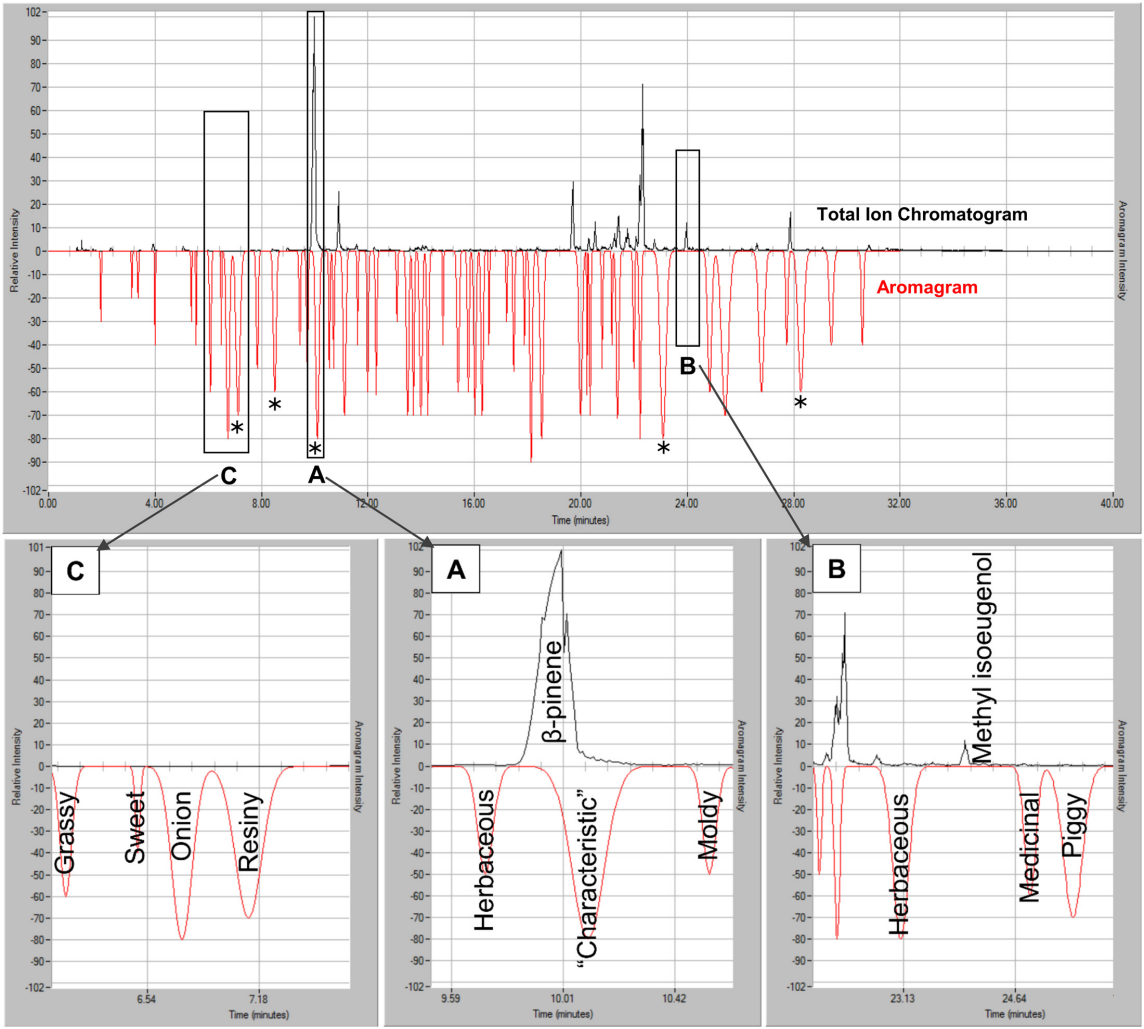
Simultaneous chemical and sensory analysis of volatiles emitted into headspace, through a duffel bag, and captured by SPME over 68 h. Total ion chromatogram (black) and aromagram, (red, inverted) of VOC emitted from marijuana in a duffel bag. An 85 μm Carboxen/PDMS SPME fiber was exposed to headspace over the duffel bag, within an overturned glass jar to capture emitted volatiles for 68 h (see S2 Fig). A total of 53 aroma events and 178 compounds were recorded (S8 Table). Aroma events # 12, 15, 46, and 51 were recorded as a ‘characteristic’ smell (i.e. (*) the aromas that most represent the overall aroma of marijuana). Outlined boxes signify (A) big chemical peak detected, smell detected; (B) chemical peak detected, no smell detected; (C) Small or no chemical peak detected, smell detected. Zoomed boxes A, B, and C identify chemical peaks and aromas.

A comparison of the total ion chromatogram generated by MS and aromagram generated by human olfaction is shown in Fig 2, illustrating simultaneous chemical and sensory detection of extracted volatiles in headspace emitted through a duffel bag.

- Box A illustrates the current understanding of compounds responsible for aroma of marijuana, showing a large chemical signal with a large olfactory intensity. There were 20 instances (9% of the identified peaks) of box A.
- Box B (black-outlined box) illustrates where a chemical present in the headspace has no perceived aroma by human nose. There were 159 instances (75% of identified peaks) of box B.
- Box C (black-outlined box) illustrates the paradigm shift of odor perception that is the main focus of this report. Chemicals having small surrogate concentration (i.e. sub-threshold detection by mass spectrometer), can register high odor impact due to OAV (1). There were 34 instances (16%) of box C.

Note the 5 “characteristic” aromas detected by human nose, with the exception of box A (identified by AMDIS as β-pinene), were not the most chemically abundant in headspace, and perceived to represent the overall aroma of marijuana. Full identification and odor characteristics of volatiles belonging to these 3 boxes are shown in S8 Table. This suggests that compounds having very small concentration in headspace of marijuana are the “needles in the haystack” of compounds responsible for overall odor of marijuana, not the most concentrated compounds as previously reported. The effects of synergistic or antagonistic effects of interactions between the components of the mixture were investigated in this study.

**Fig 3:**
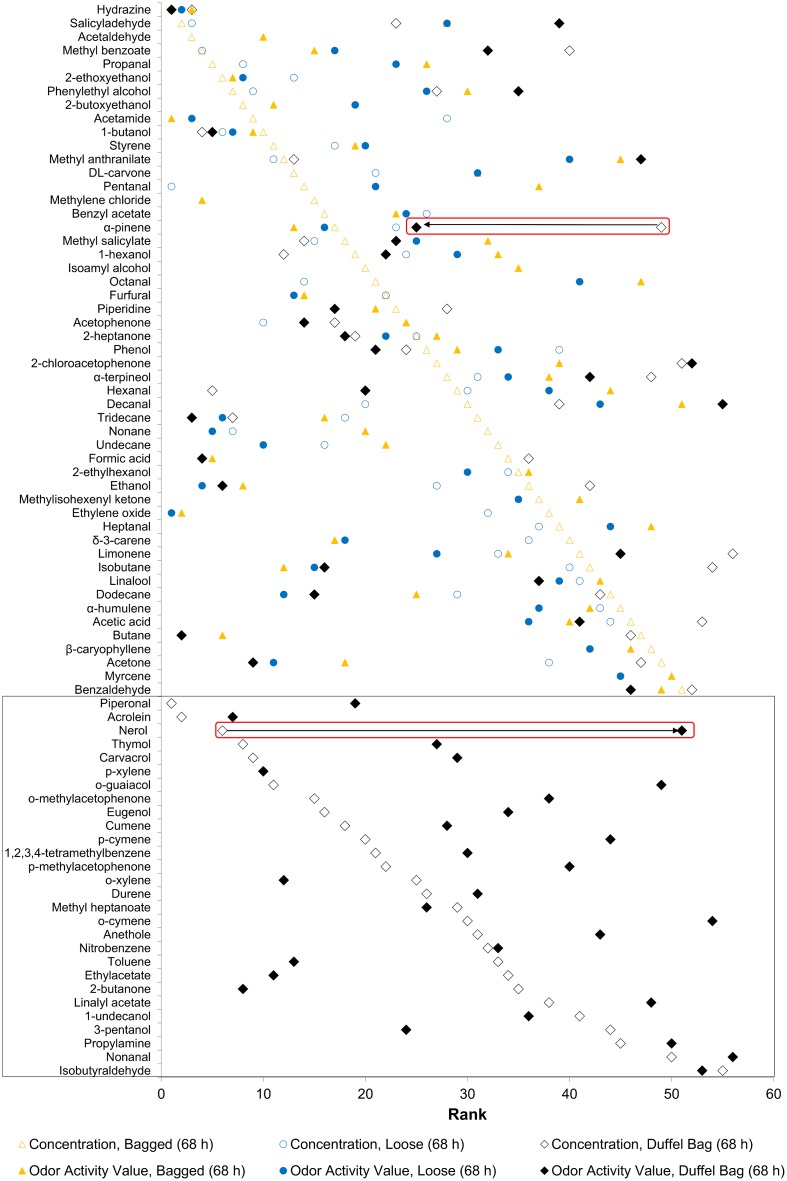
Dot plot reporting shift in rank of volatiles emitted from marijuana by surrogate concentration vs. calculated OAV. Markers represent VOC emitted from all marijuana samples and extracted from headspace by SPME over 68 h at room temperature. Compounds are ranked by surrogate concentration vs. calculated OAV using standardized ODT in air from Devos, et al. (1990) [35]. Data representing bagged and loose marijuana sampled for 68 h are similar to S5 Fig. Rank value (horizontal-axis) of 1 indicates low surrogate concentration or low OAV; rank of 60 indicates high surrogate concentration or high OAV. The general trend is a shift in rank between surrogate concentration and odor activity value. Nerol has a rank of 6 by surrogate concentration and has a rank of 51 by OAV (bottom red box); α-pinene has a rank of 49 by surrogate concentration and has a rank of 25 by OAV (top red box). Values of rank for each VOC are given in S6 Table. Compounds in the black box highlight VOC that were detected in fresh marijuana and permeated through a duffel bag, not detected or permeated in desiccated marijuana.

There were 53 aroma events identified by human panelist (S8 Table) found in the fresh marijuana sample in a duffel bag, emitted over 68 h (S2 Fig). Aroma event 36 was rated the most intense but with relatively small chemical signal from the mass detector, described as moldy, burnt, and burnt food by the panelist. The 5 “characteristic” aromas had intensities of 80, 80, 70, 60, and 60. The aroma events were ranked by aroma area (assumed to be equivalent to mass detector response); the “characteristic” aromas are within the top 15 most intense aromas. When these volatiles were ordered by surrogate concentration and compared to the OAV, we observed the same trend in rank shift (Fig 3), also observed in S3–S5 Figs, indicating that concentration and odor impact are not highly correlated (R2 < 0.1047, S7 Table).

A two-way analysis of variance (ANOVA) was performed without replication and assuming no interaction, followed by a multiple comparisons test. Normal distribution and equal variance is assumed for the analysis. The two-way ANOVA without repetition was conducted to compare the effect of packaging on VOC emitted from marijuana at 5 min, 1h, and 68 h extraction times using static headspace SPME extraction at room temperature. Just one single measurement was taken at each combination of factors; therefore, it is assumed that there were no interactions between the independent variables of sampling time and packaging. The post hoc Tukey HSD is conservative and attempts to control the overall alpha level, and is less sensitive than the ANOVA, so this could account for the 5 VOC showing no significant difference in the pairwise comparison, but indicated as significant in the ANOVA.

Fig 3 shows 79 compounds with published ODT, emitted from all marijuana samples presented thus far. It is pointed out that with missing published ODT for some compounds; this ranking by OAV is only showing information representing less than 47% of the total compounds detected by MS. Only 56 out of 178 volatiles emitted from marijuana through a cloth duffel bag, over 68 h, have published ODT. More research is warranted to establish these missing ODT. Some compounds with unpublished ODT could have extremely high or low ODT, and would alter the current rank. Shown in Fig 3, 3.7% of these 81 compounds were unique to dry marijuana in a plastic zip-top sandwich bag, 2.4% were unique to loose, dry marijuana, 34.5% were unique to fresh marijuana in a duffel bag. Highlighted Fig 3 is α-pinene, ranked 49th (high) in surrogate concentration in headspace of fresh marijuana emitted through a duffel bag, but is ranked 25th (low to mid-range) in OAV. Nerol is ranked 6th (low) in surrogate concentration in headspace of fresh marijuana emitted through duffel bag, but is ranked 51st (high) in OAV. Current research is missing the target when only the highly concentrated compounds are looked at when trying to understand odor. This data also suggests that the complete odor profile of the fresh marijuana emitted through a duffel bag is caused by a mix of compounds different from the dry marijuana (loose) or dry marijuana in a plastic zip-top sandwich bag.

### Odor impact based on OAV

**Fig 4:**
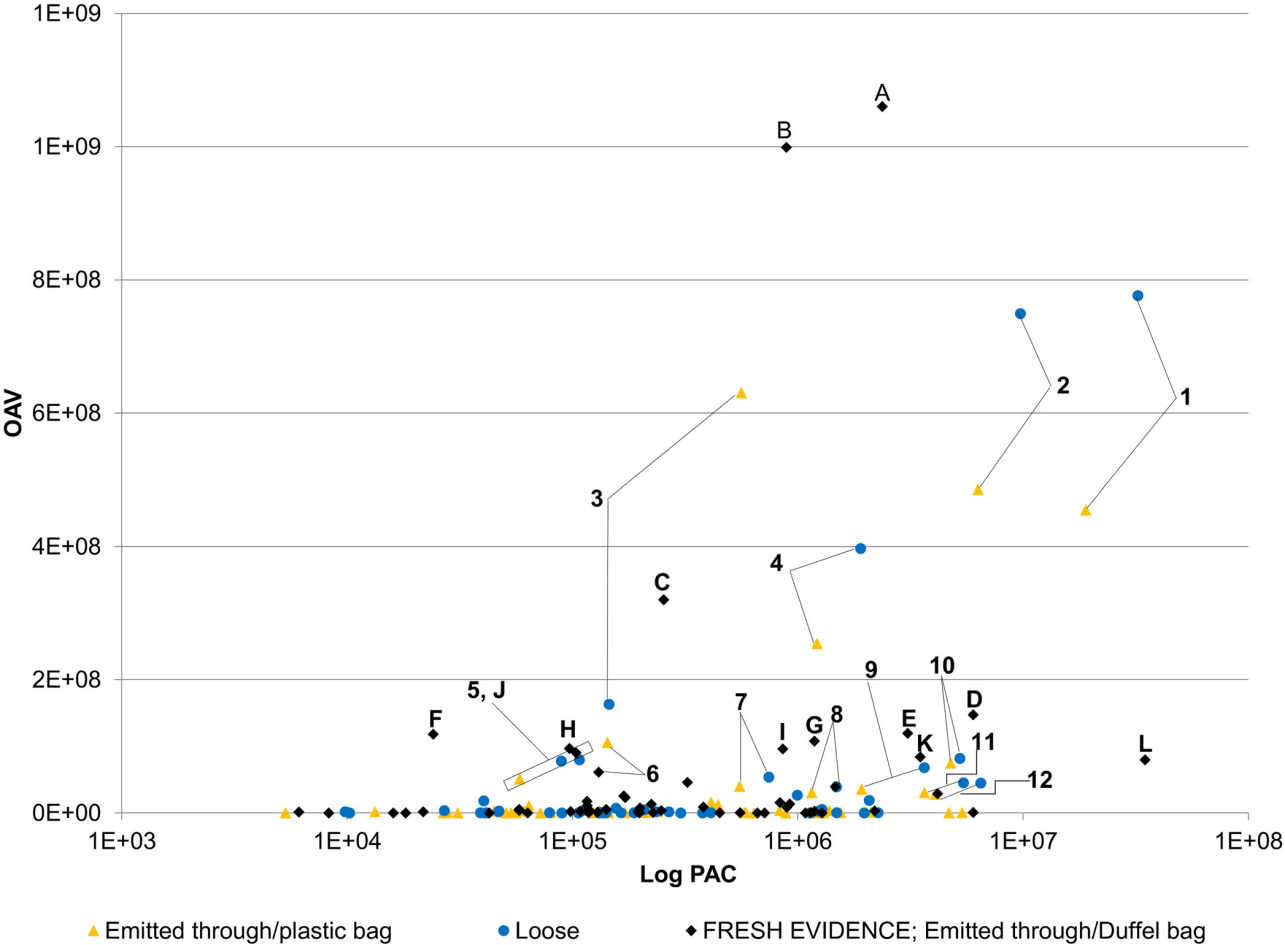
Compounds with high odor impact are not always the most abundant in concentration. Horizontal axis is peak area counts (PAC) of mass detector response on logarithmic scale, assuming equal response for all compounds. Vertical axis is calculated OAV (Eq 1) for each compound. Highly odorous compounds emitted from *loose* marijuana (blue circles) and *through packaging* (yellow triangles) over 68 h at room temperature are 1) Benzaldehyde, 2) Myrcene, 3) Decanal, 4) Heptanal, 5) Methyl anthranilate, 6) Octanal, 7) Hexanal, 8) Methylisohexenyl ketone, 9) Linalool, 10) β-caryophyllene, 11) α-humelene, 12) Acetic acid. Highly odorous compounds emitted from fresh evidence of marijuana *through a duffel bag* (black diamonds) over 68 h at room temperature are A) Nonanal, B) Decanol, C) o-cymene, D) Isobutyraldehyde, E) 1-chloroacetophenone, F) Nerol, G) Propylamine, H) o-guaiacol, I) Linalyl acetate, J) Methyl anthranilate, K) Benzaldehyde, L) Limonene.

Fig 4 illustrates that the most odorous compounds with published ODT, the further the distance on the vertical axis from zero, the more odor impact of the compound. The compounds found to be responsible for the overall aroma of dry marijuana investigated in this research, both loose and emitted through a plastic zip-top bag over 68 h are 1) Benzaldehyde, 2) Myrcene, 3) Decanal, 4) Heptanal, 5) Methyl anthranilate, 6) Octanal, 7) Hexanal, 8) Methylisohexenyl ketone, 9) Linalool, 10) β-Caryophyllene, 11) α-Humelene, and 12) Acetic acid. Highly odorous compounds with published ODT emitted from fresh marijuana through a duffel bag over 68 h are A) Nonanal, B) Decanol, C) o-Cymene, D) Isobutyraldehyde, E) 1-Chloroacetophenone, F) Nerol, G) Propylamine, H) o-Guaiacol, I) Linalyl acetate, J) Methyl anthranilate, K) Benzaldehyde, L) Limonene. Top ranked volatiles (by OAV) do not agree with what is currently known as key odorous compounds responsible for the smell of marijuana [1–6]. Also, results of this research indicate the key odorous compounds responsible for the smell of marijuana are different between old, desiccated marijuana and fresh marijuana.

## Conclusions

Odorous compounds emitted from marijuana were identified using MDGC-MS coupled with simultaneous human olfaction. Over 200 compounds are being added to the list of what is currently known to be emitted from illicitly packaged marijuana. It is suggested that newly packaged marijuana (i.e. packaged or sitting in a room for 5 min) would have a different aroma profile than marijuana that has been stored for a longer period (i.e. packaged or sitting in a room for 68 h) due to the increased number of chemical peaks detected by MDGCMS-O (~20 compounds to ~100 compounds, respectively). Overall odor of marijuana due to compounds emitted is time dependent, but effects of plastic zip-top sandwich bag or cloth duffel bag packaging on compound surrogate concentration were not significant (p <0.05). When simultaneous chemical and sensory analysis was used to analyze headspace volatiles of marijuana emitted through a duffel bag, 9% of the chemicals detected by MS had an associated aroma, 75% of the chemicals detected did not have an aroma detected, and 16% registered low or no chemical signal but an aroma was detected. This phenomenon can be explained by taking into account OAV. The application of OAV to forensic odor is being proposed as a novel approach to investigating forensic odor. More work is needed to establish ~55% of missing ODT and ~41% missing odor description. This reports suggests that highly odorous compounds are not necessarily the most concentrated compounds in headspace. This is the first reported instance of using MDGC-MS tandem simultaneous olfactometry by human nose to characterize the volatiles in the total aroma profile emitted from marijuana in the context of non-destructive, through-packaging analysis of evidence. Shifting the focus to using OAV to evaluate total odor, instead of analyzing the most concentrated compounds, can help forensic investigators understand cadaver odor, drug odor, explosives odor, etc. This draws attention to how training a drug detection dog, handlers, and other law enforcement officers to a handful of compounds does not cover the gamut of VOC found in different conditions of marijuana for illicit distribution.

## Supporting Information

S1 Fig Static headspace sampling of VOC emitted at room temperature from illegal street drugs. (Samples from left to right). The SPME fiber is exposed and sampling in between the evidence bag and original packaging of cocaine. ~1 gram of air-dried marijuana in a zip-top plastic sandwich bag. ~1 gram of air-dried marijuana, loose in the jar. Methamphetamine in a beaker. Holes were predrilled into the metal mason jar lids, and fitted with a half-hole septa as the SPME sampling port. All jars, lids, rings, and septa were pre-cleaned and baked out in 110°C oven overnight to desorb interfering VOC. Pre-conditioned SPME fibers were pre-cleaned prior to sampling by desorbing in a 270°C GC injection port under flow of nitrogen, retracted, and wrapped in aluminum foil for transport. Sample loaded fibers were transported back to the lab wrapped in clean foil, placed in a clean jar with intact metal lid, and kept in a cooler with reusable ice packs.
(PDF)

S2 Fig Static headspace sampling of VOC at room temperature from marijuana emitted though a duffel bag. A US military-style duffel bag containg ~50 kg of marijuana was siezed and tagged as evidence. The SPME fiber was exposed and propped up by a metal binder clip, inside an over-turned, pre-cleaned 16 oz glass mason jar. This ad hoc apparatus created a headspace sampling chamber to collect VOC emitted from the marijuana and through the duffel bag over a period of 68 h. The fiber was transported back to the lab for analysis as described in the caption of S1 Fig.
(PDF)

S3 Fig Dot plot illustrating hierarchy of volatiles emitted from marijuana using surrogate concentration and calculated OAV from published ODT at 5 min. Open markers represent the rank of the volatile based on surrogate concentration. Closed markers represent the rank of the volatile based on OAV. Horizontal axis reads from left to right, indicating least to most concentrated/odor active rank. Rank number is provided above and below markers for ease of reading. The general inference is a shift in rank based on OAV. Compounds with low detection thresholds tend to rank higher in OAV than rank of surrogate concentration in headspace, a relationship shown by Eq 1. Blue box-outlined markers indicate volatiles detected in unpackaged marijuana and not detected by through-package sampling.
(PDF)

S4 Fig Dot plot illustrating hierarchy of volatiles emitted from marijuana using surrogate concentration and calculated OAV from published ODT at 1 h. Open markers represent the rank of the volatile based on surrogate concentration. Closed markers represent the rank of the volatile based on OAV. Horizontal axis reads from left to right, indicating least to most concentrated/odor active rank. Rank number is provided above and below markers for ease of reading. The general inference is a shift in rank based on OAV. Compounds with low detection thresholds tend to rank higher in OAV than rank of surrogate concentration in headspace, a relationship shown by Eq 1. Blue box-outlined markers indicate volatiles detected in unpackaged marijuana and not detected by through-package sampling.
(PDF)

S5 Fig Dot plot illustrating hierarchy of volatiles emitted from marijuana using surrogate concentration and calculated OAV from published ODT at 68 h. Open markers represent the rank of the volatile based on surrogate concentration. Closed markers represent the rank of the volatile based on OAV. Horizontal axis reads from left to right, indicating least to most concentrated/odor active rank. Rank number is provided above and below markers for ease of reading. The general inference is a shift in rank based on OAV. Compounds with low detection thresholds tend to rank higher in OAV than rank of surrogate concentration in headspace, a relationship shown by Eq 1. Blue box-outlined markers indicate volatiles detected in unpackaged marijuana and not detected by through-package sampling.
(PDF)

S1 Table Legal cases based on probable cause for search and seizure. (PDF)

S2 Table Summary of VOC emitted from marijuana though packaging into headspace and captured by SPME during 5 min, 1h, 68 h static sampling at room temperature. (PDF)

S3 Table Summary of VOC emitted from unpackaged marijuana into headspace and captured by SPME during 5 min, 1h, 68 h static sampling at room temperature. (PDF)

S4 Table Summary of F-statistics and p-values from two-way analysis of variance comparing the effect of packaging on VOC emitted from marijuana at 5 min, 1 h, and 68 h extraction times. (PDF)

S5 Table Wilcoxon signed rank test of paired samples. (PDF)

S6 Table Hierarchy of volatile compounds with published ODT, emitted from marijuana, through packaging over 68 h. (PDF)

S7 Table Correlation coefficients between surrogate concentration and odor impact of volatile compounds emitted from marijuana. (PDF)

S8 Table Identification of VOC emitted though cloth duffel bag in headspace of marijuana sample, and captured by SPME over 68 h. (PDF)
