# Supplementary material for: Characterizing the Smell of Marijuana by Odor Impact of Volatile Compounds: An Application of Simultaneous Chemical and Sensory Analysis
Source: PLoS One. 2015 Dec 10;10(12):e0144160. doi: 10.1371/journal.pone.0144160 (PMC4684335; doi:10.1371/journal.pone.0144160)
Supplement: S1 Fig — (Samples from left to right). The SPME fiber is exposed and sampling in between the evidence bag and original packaging of cocaine. ~1 gram of air-dried marijuana in a zip-top plastic sandwich bag. ~1 gram of air-dried marijuana, loose in the jar. Methamphetamine in a beaker. Holes were predrilled into the metal mason jar lids, and fitted with a half-hole septa as the SPME sampling port. All jars, lids, rings, and septa were pre-cleaned and baked out in 110°C oven overnight to desorb interfering VOC. Pre-conditioned SPME fibers were pre-cleaned prior to sampling by desorbing in a 270°C GC injection port under flow of nitrogen, retracted, and wrapped in aluminum foil for transport. Sample loaded fibers were transported back to the lab wrapped in clean foil, placed in a clean jar with intact metal lid, and kept in a cooler with reusable ice packs. (PDF) [file pone.0144160.s001.pdf]

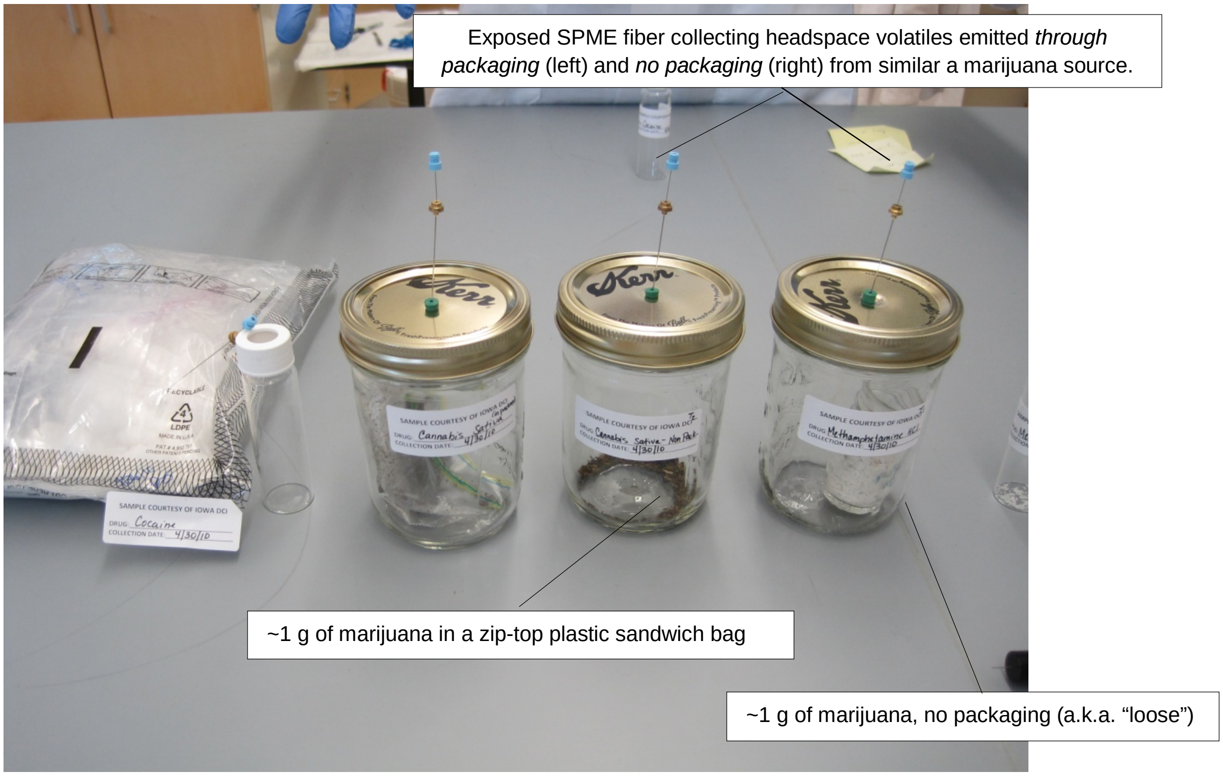

Figure S1. Static headspace sampling of VOC emitted at room temperature from illegal street drugs. (Samples from left to right. The SPME fiber is exposed and sampling in between the evidence bag and original packaging of cocaine. ~1 gram of air-dried marijuana in a zip-top plastic sandwich bag. ~1 gram of air-dried marijuana, loose in the jar. Methamphetamine in a beaker. Holes were predrilled into the metal mason jar lids, and fitted with a half-hole septa as the SPME sampling port. All jars, lids, rings, and septa were pre-cleaned and baked out in 110 °C oven overnight to desorb interfering VOC. Pre-conditioned SPME fibers were pre-cleaned prior to sampling by desorbing in a 270 °C GC injection port under flow of nitrogen, retracted, and wrapped in aluminum foil for transport. Sample loaded fibers were transported back to the lab wrapped in clean foil, placed in a clean jar with intact metal lid, and kept in a cooler with reusable ice packs.
