# Supplementary material for: Characterizing the Smell of Marijuana by Odor Impact of Volatile Compounds: An Application of Simultaneous Chemical and Sensory Analysis
Source: PLoS One. 2015 Dec 10;10(12):e0144160. doi: 10.1371/journal.pone.0144160 (PMC4684335; doi:10.1371/journal.pone.0144160)
Supplement: S2 Fig — A US military-style duffel bag containg ~50 kg of marijuana was siezed and tagged as evidence. The SPME fiber was exposed and propped up by a metal binder clip, inside an over-turned, pre-cleaned 16 oz glass mason jar. This ad hoc apparatus created a headspace sampling chamber to collect VOC emitted from the marijuana and through the duffel bag over a period of 68 h. The fiber was transported back to the lab for analysis as described in the caption of S1 Fig. (PDF) [file pone.0144160.s002.pdf]

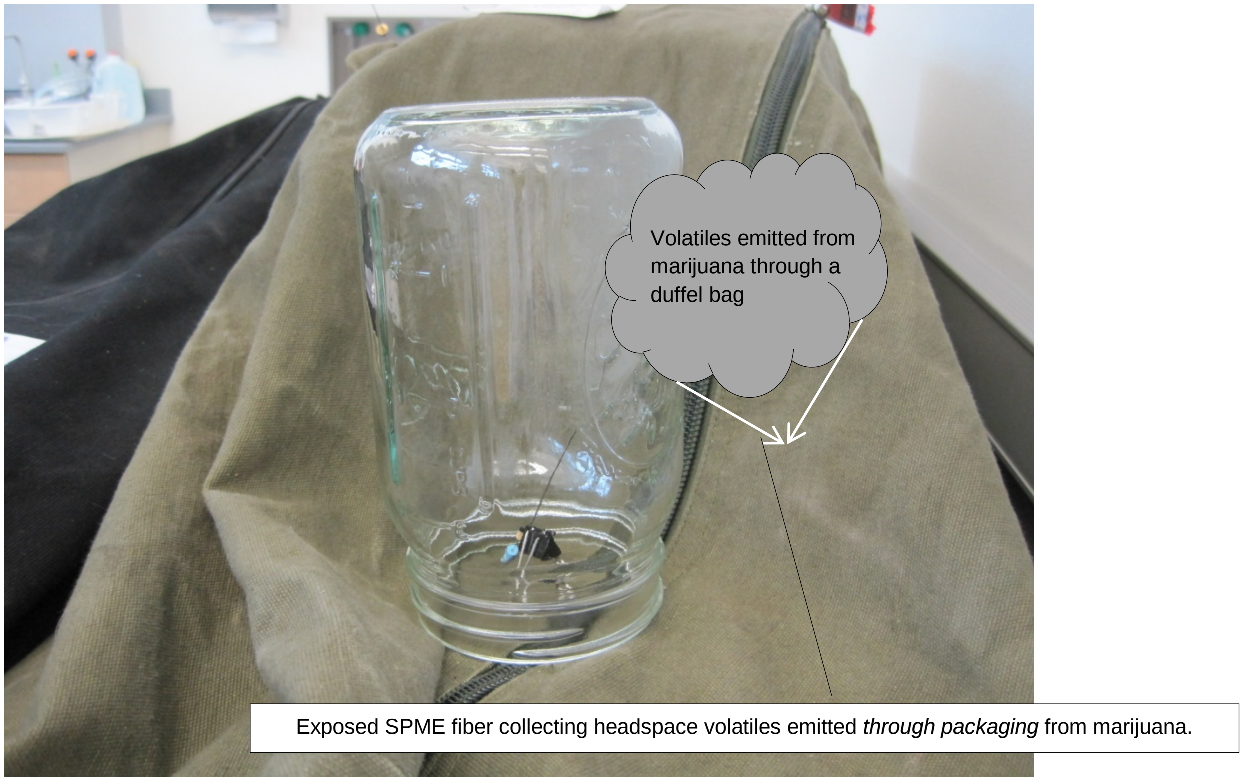


**Figure S2. Static headspace sampling of VOC at room temperature from marijuana emitted though a duffel bag.** A US military-style duffel bag containg ~50 kg of marijuana was siezed and tagged as evidence. The SPME fiber was exposed and propped up by a metal binder clip, inside an over-turned, pre-cleaned 16 oz glass mason jar. This ad hoc apparatus created a headspace sampling chamber to collect VOC emitted from the marijuana and through the duffel bag over a period of 68 h. The fiber was transported back to the lab for analysis as described in the caption of SI Figure 1.
