# Supplementary material for: Characterizing the Smell of Marijuana by Odor Impact of Volatile Compounds: An Application of Simultaneous Chemical and Sensory Analysis
Source: PLoS One. 2015 Dec 10;10(12):e0144160. doi: 10.1371/journal.pone.0144160 (PMC4684335; doi:10.1371/journal.pone.0144160)
Supplement: S4 Fig — Open markers represent the rank of the volatile based on surrogate concentration. Closed markers represent the rank of the volatile based on OAV. Horizontal axis reads from left to right, indicating least to most concentrated/odor active rank. Rank number is provided above and below markers for ease of reading. The general inference is a shift in rank based on OAV. Compounds with low detection thresholds tend to rank higher in OAV than rank of surrogate concentration in headspace, a relationship shown by Eq 1. Blue box-outlined markers indicate volatiles detected in unpackaged marijuana and not detected by through-package sampling. (PDF) [file pone.0144160.s004.pdf]

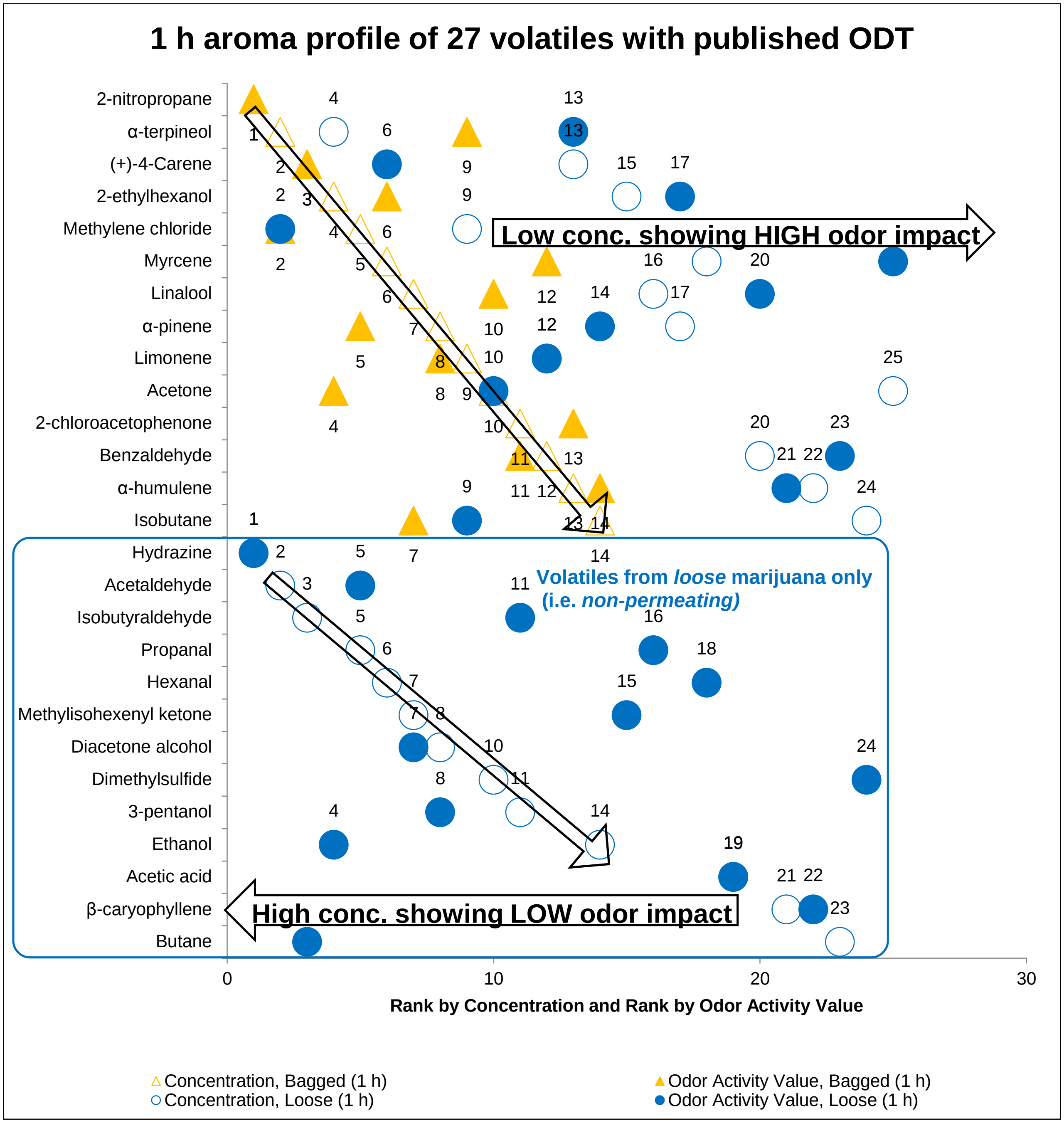

Figure S4. Dot plot illustrating hierarchy of volatiles emitted from marijuana using surrogate concentration and calculated OAV from published ODT at 1 h. Open markers represent the rank of the volatile based on surrogate concentration. Closed markers represent the rank of the volatile based on OAV. Horizontal axis reads from left to right, indicating least to most concentrated/odor active rank. Rank number is provided above and below markers for ease of reading. The general inference is a shift in rank based on OAV. Compounds with low detection thresholds tend to rank higher in OAV than rank of surrogate concentration in headspace, a relationship shown by Eq. 1. Blue box-outlined markers indicate volatiles detected in unpackaged marijuana and not detected by through-package sampling.
