# Supplementary material for: Characterizing the Smell of Marijuana by Odor Impact of Volatile Compounds: An Application of Simultaneous Chemical and Sensory Analysis
Source: PLoS One. 2015 Dec 10;10(12):e0144160. doi: 10.1371/journal.pone.0144160 (PMC4684335; doi:10.1371/journal.pone.0144160)
Supplement: S1 Table — (PDF) [file pone.0144160.s006.pdf]

Table S1. Legal cases based on probable cause for search and seizure.

**Legal Case**	**Ruling**
Payton v. New York, 445 U.S. 573 (1980)	A warrantless search **inside your home** is reasonable if there is *probable cause* or exigent circumstances
Maryland v. Macon, 472 U.S. 463 (1985)	A warrantless search **inside your home** is reasonable if items are in plain view
Davis v. United States, 328 U.S. 582 (1946)	A warrantless search **inside your home** is reasonable if an officer is given consent
United States v. Robinson, 414 U.S. 218 (1973)	A warrantless search **inside your home** is reasonable if the search is incident to a lawful arrest
New Jersey v. TLO, 469, U.S. 325 (1985)	A warrantless search of **a student** under the authority of school officials is reasonable
Arizona v. Gant, 129 S. Ct. 1710 (2009)	A **vehicle** may be searched in the area where evidence might be found, if there is *probable cause* of the vehicle containing evidence of criminal activity
Illinois v. Cabales, 543 U.S. 405 (2005)	A narcotics detection dog may walk around the exterior of the **vehicle** in a valid traffic stop without reasonable or explainable suspicion
United States of America v. Harris, 846 F. Supp. 121 (1994)	Police officers used the smell of marijuana as *probable* cause to search a **vehicle** only to find cocaine in the car, but no marijuana.
