# Supplementary material for: Characterizing the Smell of Marijuana by Odor Impact of Volatile Compounds: An Application of Simultaneous Chemical and Sensory Analysis
Source: PLoS One. 2015 Dec 10;10(12):e0144160. doi: 10.1371/journal.pone.0144160 (PMC4684335; doi:10.1371/journal.pone.0144160)
Supplement: S2 Table — (PDF) [file pone.0144160.s007.pdf]

Table S2. Summary of VOC emitted from marijuana though packaging into headspace and captured by SPME during 5 min, 1h, 68 h static sampling at room temperature.

							Published Descriptors		Published ODT (ppm)			
Compound	CAS	Ext. Time	Peak #	RT (min)	Models	Net % Match	Flavornet[1]	TGSC[2]	LRI & Odour Database[3]	Devos, et al. [4]	Surrogate Conc. (PAC)	OAV
Ethylene oxide	75-21-8	5 min	1	1.06	4: 44 45 132 46	66				8.51E+02	1.42E+06	1.66E+03
		68 h	1	1.07	2: 44 45	66				8.51E+02	1.22E+06	1.43E+03
2-nitropropane	79-46-9	5 min	2	1.12	2: 43 58	72				7.24E+00	1.24E+04	1.71E+03
		1 h	1	1.12	2: 41 43	75				7.24E+00	6.30E+03	8.69E+02
Isobutane	75-28-5	1 h	2	1.23	10: 43 42 41 39 72 55 50 73 71 58	84				1.00E+01	7.18E+06	7.18E+05
		68 h	2	1.24	6: 43 42 41 39 53 50	85				1.00E+01	1.55E+06	1.55E+05
Isobutyraldehyde	78-84-2	5 min	3	1.23	9: 43 42 41 57 39 55 51 38 37	78	Pungent, Malt, Green	Spicy		4.07E-02	1.84E+06	4.51E+07
Methyl mercaptan	74-93-1	68 h	3	1.27	2: 47 48	87	Sulfur, Gasoline, Garlic	Decomposing, Cabbage, Garlic			1.86E+04	
Acetaldehyde	75-07-0	68 h	4	1.28	2: 44 43	80	Pungent, Ether	Pungent, Ethereal, Aldehydic, Fruity		1.86E-01	2.67E+04	1.44E+05
Isoprene	78-79-5	1 h	3	1.33	1: 67	71					1.73E+04	
		68 h	5	1.34	TIC	88					1.38E+04	
4-methyldecane	2847-72-5	5 min	4	1.39	9: 43 42 71 41 57 39 56 85 51	67					4.31E+05	
		1 h	5	1.40	10: 39 57 55 41 86 53 69 38 52 67	66					1.35E+06	
2-methylpentane	107-83-5	5 min	5	1.40	2: 57 86	97					4.02E+05	
		1 h	4	1.40	8: 43 71 42 41 57 50 56 86	98					1.54E+06	
		68 h	6	1.40	7: 43 42 41 71 67 70 72	96					2.15E+05	
2,3,4-trimethylpentane	565-75-3	68 h	7	1.41	3: 43 39 71	80					1.77E+05	
Isocyanatomethane	624-83-9	5 min	6	1.44	2: 39 70	68					3.68E+04	

							Published Descriptors		Published ODT (ppm)			
Compound	CAS	Ext. Time	Peak #	RT (min)	Models	Net % Match	Flavornet[1]	TGSC[2]	LRI & Odour Database[3]	Devos, et al. [4]	Surrogate Conc. (PAC)	OAV
		68 h	9	1.45	3: 57 56 67	78					3.85E+04	
Ethylenimine	151-56-4	5 min	7	1.45	3: 42 43 55	76					1.94E+04	
		1 h	7	1.53	1: 41	70					5.22E+04	
2-methylaziridine	75-55-8	5 min	8	1.45	TIC	83					3.61E+04	
		68 h	8	1.45	1: 56	78					4.85E+04	
3-methylpentane	96-14-0	5 min	9	1.45	3: 56 57 53	91					1.05E+05	
		1 h	6	1.45	5: 57 56 55 58 86	98					4.24E+05	
		68 h	10	1.46	TIC	90					2.24E+04	
Propanal	123-38-6	68 h	11	1.57	TIC	75	Solvent, Pungent	Earthy, Alcohol, Wine, Whiskey, Cocoa, Nutty		2.69E-02	2.79E+04	1.04E+06
Butane	106-97-8	68 h	13	1.65	8: 43 58 42 39 53 57 41 44	83				2.04E+02	4.69E+06	2.30E+04
1-Propanamine, 3-dibenzo[b,e]thiepin-11(6H)-ylidene-N,N-dimethyl-, S-oxide	1447-71-8	68 h	14	1.66	4: 37 59 60 55	73					3.29E+05	
Acetone	67-64-1	5 min	10	1.66	2: 58 43	89		Solvent		1.45E+01	7.49E+04	5.18E+03
		1 h	8	1.65	5: 43 42 39 41 37	96		Solvent		1.45E+01	4.78E+05	3.30E+04
		68 h	12	1.65	TIC	99		Solvent		1.45E+01	5.35E+06	3.70E+05
Methacrolein	78-85-3	68 h	15	2.13	TIC	95		Wild hyacinth foliage			1.40E+05	
Formic acid	64-18-6	68 h	16	2.33	3: 45 72 42	69		Acetic		2.82E+01	6.40E+05	2.27E+04
Ethanol	64-17-5	5 min	11	2.34	2: 45 43	91	Sweet	Alcoholic		2.88E+01	7.27E+04	2.52E+03
		68 h	17	2.34	6: 45 43 46 72 42 41	93	Sweet	Alcoholic		2.88E+01	8.83E+05	3.06E+04
Methylene chloride	75-09-2	5 min	12	2.42	4: 84 51 49 86	93				2.82E+01	4.51E+04	1.60E+03
		1 h	9	2.43	2: 47 49	98				2.82E+01	2.81E+05	9.98E+03
		68 h	18	2.43	3: 84 49 51	94				2.82E+01	7.21E+04	2.56E+03
Pentanal	110-62-3	68 h	19	3.66	TIC	84	Almond, Malt, Pungent	Fermented		6.03E-03	6.40E+04	1.06E+07

Compound	CAS	Ext. Time	Peak #	RT (min)	Models	Net % Match	Published Descriptors: Flavornet[1]	Published Descriptors: TGSC[2]	Published ODT (ppm): LRI & Odour Database[3]	Published ODT (ppm): Devos, et al. [4]	Surrogate Conc. (PAC)	OAV
tert-butanol	75-65-0	5 min	13	3.91	1: 59	67		Camphor			2.32E+04	
		68 h	23	3.94	2: 33 41	74		Camphor			5.49E+04	
Ethylenediamine	107-15-3	5 min	14	3.92	TIC	73					1.04E+05	
		68 h	21	3.93	TIC	73					1.13E+05	
1,1-dimethyl-hydrazine	57-14-7	5 min	15	3.93	TIC	79					5.44E+04	
		68 h	20	3.92	TIC	79					3.19E+05	
Hydrazine	302-01-2	5 min	16	3.95	1: 33	76		Ammoniacal		3.00E+00	1.58E+03	5.26E+02
		68 h	22	3.93	1: 33	77				3.00E+00	5.33E+03	1.78E+03
Hexanal	66-25-1	68 h	24	5.99	4: 44 42 67 40	96	Grass, Tallow, Fat	Green		1.38E-02	5.52E+05	4.00E+07
1-butanol	71-36-3	68 h	25	6.12	TIC	93	Medicine, Fruit	Fermented		4.90E-01	5.54E+04	1.13E+05
Nonane	111-84-2	68 h	26	6.70	11: 41 71 85 70 39 129 83 42 98 53 45	95	Alkane	Gasoline		1.26E+00	5.78E+05	4.59E+05
Sabinene	3387-41-5	5 min	17	7.90	4: 93 79 107 106	82	Pepper, Turpentine, Wood	Woody			6.25E+04	
α-phellandrene	99-83-2	5 min	19	7.90	TIC	87	Turpentine, Mint, Spice	Terpenic			5.43E+04	
		1 h	12	7.92	13: 94 91 93 55 51 136 92 79 121 77 108 103 122	90	Turpentine, Mint, Spice	Terpenic			8.87E+04	
		68 h	27	7.93	11: 93 91 55 92 94 79 41 136 77 107 105	86	Turpentine, Mint, Spice	Terpenic			2.28E+05	
α-pinene	80-56-8	5 min	18	7.90	TIC	85	Pine, Turpentine	Herbal		6.92E-01	5.43E+04	7.85E+04
		1 h	13	7.93	8: 93 81 68 107 43 105 95 78	93	Pine, Turpentine	Herbal		6.92E-01	3.65E+05	5.28E+05
		68 h	28	7.93	TIC	92	Pine, Turpentine	Herbal		6.92E-01	1.24E+05	1.80E+05
γ-terpinene	99-85-4	5 min	20	7.90	4: 93 53 136 41	74	Gasoline, Turpentine	Terpenic			9.27E+04	
2-isopropenyl-3-methylpyrazine	145984-65-2	5 min	21	7.90	1: 134	67					7.52E+03	
(+)-4-Carene	29050-33-7	1 h	10	7.91	5: 105 119 121 80 136	67			4.00E+00		7.28E+04	1.82E+04

Compound	CAS	Ext. Time	Peak #	RT (min)	Models	Net % Match	Published Descriptors: Flavornet[1]	Published Descriptors: TGSC[2]	Published ODT (ppm): LRI & Odour Database[3]	Published ODT (ppm): Devos, et al. [4]	Surrogate Conc. (PAC)	OAV
Betahistine	5638-76-6	1 h	11	7.91	8: 136 93 80 43 41 106 65 94	70					5.56E+05	
2-heptanone	110-43-0	68 h	29	8.45	TIC	93	Soap	Cheesy		1.41E-01	2.14E+05	1.51E+06
Isoamyl alcohol	123-51-3	68 h	30	8.61	4: 67 53 39 85	67	Whiskey, Malt, Burnt	Fusel oil, Alcoholic, Whiskey, Fruity, Banana		4.47E-02	1.41E+05	3.17E+06
Heptanal	111-71-7	68 h	31	8.62	13: 42 69 71 55 39 81 86 45 96 85 53 54 65	97	Fat, Citrus, Rancid	Green		4.79E-03	1.22E+06	2.54E+08
4-methylpyrimidine	3438-46-8	68 h	32	9.06	4: 94 67 51 42	91					1.75E+05	
4-pyridinamine	504-24-5	68 h	33	9.06	TIC	88					7.41E+04	
1,3,5-triazine-2,4,6-triamine	108-78-1	5 min	22	9.27	1: 126	67					2.43E+03	
Styrene	100-42-5	68 h	34	9.45	4: 103 78 51 83	92	Balsamic, Gasoline	Balsamic		1.45E-01	5.62E+04	3.89E+05
Myrcene	123-35-3	1 h	14	9.93	TIC	92	Balsamic, Must, Spice	Peppery, Terpene, Spicy, Balsam, Plastic	1.30E-02		2.88E+05	2.22E+07
		68 h	35	9.94	17: 94 137 66 82 89 42 115 70 119 54 61 107 122 76 57 56 62	94	Balsamic, Must, Spice	Peppery, Terpene, Spicy, Balsam, Plastic	1.30E-02		6.31E+06	4.85E+08
β-pinene	18172-67-3	68 h	36	9.97	17: 70 121 43 54 38 56 122 62 106 89 83 134 61 137 76 75 120	97	Pine, Resin, Turpentine	Terpenic			1.67E+07	

Compound	CAS	Ext. Time	Peak #	RT (min)	Models	Net % Match	Published Descriptors: Flavornet[1]	Published Descriptors: TGSC[2]	Published ODT (ppm): LRI & Odour Database[3]	Published ODT (ppm): Devos, et al. [4]	Surrogate Conc. (PAC)	OAV
Dimethylpyrazine	123-32-0	68 h	37	10.63	TIC	71	Cocoa, Roasted nut, Roast beef, Medicine	Cocoa, Roasted nuts, Roast Beef, Woody, Grass, Medical			7.94E+03	
1-hexanol	111-27-3	68 h	38	10.74	4: 56 69 42 84	85	Resin, Flower, Breen	Herbal		4.37E-02	1.38E+05	3.15E+06
Camphene	79-92-5	1 h	16	10.89	12: 91 53 67 65 121 107 80 105 93 77 41 95	87	Camphor	Woody			4.38E+05	
		68 h	40	10.91	TIC	89	Camphor	Woody			1.38E+06	
Limonene	138-86-3	5 min	23	10.89	3: 93 67 68	68	Lemon, Orange	Citrus		4.37E-01	1.42E+04	3.26E+04
		1 h	15	10.89	12: 91 53 67 65 121 107 80 105 93 77 41 95	92	Lemon, Orange	Citrus		4.37E-01	4.38E+05	1.00E+06
		68 h	39	10.91	TIC	95	Lemon, Orange	Citrus		4.37E-01	1.38E+06	3.17E+06
Piperidine	110-89-4	68 h	41	11.20	2: 44 57	67		Animal		3.72E-01	1.71E+05	4.61E+05
Octanal	124-13-0	68 h	42	11.21	TIC	89	Fat, Soap, Lemon, Green	Aldehydic, Waxy, Citrus, Orange peel, Green, Fatty		1.35E-03	1.43E+05	1.06E+08
m-cymene	535-77-3	68 h	43	11.38	TIC	91					3.47E+04	
Methylisohexenyl ketone	110-93-0	68 h	44	11.54	TIC	95	Pepper, Mushroom, Rubber	Citrus		3.80E-02	1.15E+06	3.04E+07
δ-3-carene	13466-78-9	68 h	45	11.63	TIC	97	Lemon, Resin	Citrus	4.00E+00		1.30E+06	3.25E+05
1-butoxy-2-propanol	5131-66-8	68 h	46	11.77	2: 57 75	67					5.72E+04	
2-butoxyethanol	111-76-2	68 h	47	11.77	TIC	83				3.39E-01	5.08E+04	1.50E+05
Undecane	1120-21-4	68 h	48	11.89	8: 42 84 85 156 113 55 112 39	95	Alkane			1.17E+00	5.89E+05	5.01E+05
Acetic acid	64-19-7	68 h	49	12.23	6: 45 43 60 42 41 61	99	Sour	Acidic		1.45E-01	4.07E+06	2.82E+07

Compound	CAS	Ext. Time	Peak #	RT (min)	Models	Net % Match	Published Descriptors: Flavornet[1]	Published Descriptors: TGSC[2]	Published ODT (ppm): LRI & Odour Database[3]	Published ODT (ppm): Devos, et al. [4]	Surrogate Conc. (PAC)	OAV
Furfural	98-01-1	68 h	50	12.70	6: 95 96 67 97 38 37	97	Bread, Almond, Sweet	Sweet, Woody, Almond, Baked bread		7.76E-01	1.44E+05	1.86E+05
Citronellolformate	105-85-1	68 h	51	13.09	11: 69 41 105 65 77 54 51 138 96 42 81	70		Floral			2.96E+05	
1,3-dichlorobenzene	541-73-1	68 h	52	13.16	8: 111 50 113 74 149 55 112 75	99					4.74E+05	
2-ethylhexanol	104-76-7	1 h	17	13.81	TIC	87	Rose, Green	Citrus		2.45E-01	1.48E+05	6.05E+05
		68 h	53	13.83	15: 57 41 121 43 71 136 70 83 84 55 98 39 69 81 53	86	Rose, Green	Citrus		2.45E-01	8.30E+05	3.38E+06
5-methylindane	874-35-1	68 h	54	13.90	9: 132 92 115 131 73 91 133 65 128	82					4.21E+05	
2-ethenyl-1,3-dimethylbenzene	2039-90-9	68 h	55	13.91	7: 115 45 102 129 114 82 39	87					2.52E+05	
Benzaldehyde	100-52-7	1 h	18	14.08	TIC	98	Almond, Burnt sugar	Fruity		4.17E-02	8.98E+05	2.15E+07
		68 h	56	14.06	11: 106 105 77 51 52 76 53 39 38 62 36	99	Almond, Burnt sugar	Fruity		4.17E-02	1.90E+07	4.55E+08
2-chloro acetophenone	532-27-4	5 min	24	14.12	2: 77 105	75				2.57E-02	4.00E+03	1.56E+05
		1 h	19	14.08	6: 105 51 77 52 78 63	78				2.57E-02	6.57E+05	2.56E+07
		68 h	57	14.07	6: 57 60 49 43 61 86	77				2.57E-02	4.11E+05	1.60E+07
Dodecane	112-40-3	68 h	58	14.21	20: 57 55 41 59 122 83 112 72 113 93 70 170 98 67 127 95 97 193 171 58	70	Alkane	Alkane		2.04E+00	2.11E+06	1.03E+06
tert-butyl-benzene	98-06-6	68 h	59	14.49	7: 92 117 119 120 115 131 66	89					1.24E+05	

Compound	CAS	Ext. Time	Peak #	RT (min)	Models	Net % Match	Published Descriptors		Published ODT (ppm)		Surrogate Conc. (PAC)	OAV
							Flavornet[1]	TGSC[2]	LRI & Odour Database[3]	Devos, et al. [4]		
Linalool	78-70-6	1 h	20	15.12	TIC	91	Flower, Lavender	Floral		5.37E-02	3.31E+05	6.16E+06
		68 h	60	15.14	19: 71 93 43 41 69 80 121 67 82 65 83 72 81 111 107 105 136 39 79	98	Flower, Lavender	Floral		5.37E-02	1.92E+06	3.57E+07
cis-2-pinanol	4948-29-2	1 h	21	15.43	TIC	72		Herbal			1.30E+04	
Benzonitrile	100-47-0	68 h	61	15.46	TIC	87	Rancid, Sweet				7.23E+04	
α-ionol	25312-34-9	68 h	62	15.67	TIC	81		Ionone, Tropical, Sweet, Floral, Violet, Woody			8.15E+05	
Fenchyl alcohol	1632-73-1	1 h	22	15.72	TIC	78	Camphor	Camphor, Borneol, Pine, Woody, Dry, Sweet, Lemon			5.65E+04	
2-ethoxyethanol	110-80-5	68 h	63	15.78	2: 45 60	69				1.23E+00	3.10E+04	2.52E+04
Decanal	112-31-2	68 h	64	15.90	12: 43 82 57 71 83 56 70 95 39 72 128 97	93	Soap, Orange peel, Tallow	Aldehydic		8.91E-04	5.62E+05	6.30E+08
Methyl heptadienone	1604-28-0	68 h	65	15.91	17: 55 41 71 81 43 54 77 42 110 44 56 85 96 79 53 65 128	72		Cinnamon, Coconut, Spice, Woody, Sweet, Weedy			4.69E+05	
Methyl benzoate	93-58-3	68 h	66	16.25	4: 115 51 77 130	75	Prune, Lettuce, Herb, Sweet	Phenolic		1.07E-01	2.73E+04	2.55E+05
Tridecane	629-50-5	68 h	67	16.38	15: 57 43 55 85 141 84 56 99 58 86 69 83 127 39 53	92	Alkane	Alkane		2.14E+00	5.78E+05	2.70E+05
Acetophenone	98-86-2	68 h	68	16.50	5: 77 226 51 163 50	92	Musty, Flower, Almond	Floral		3.63E-01	2.09E+05	5.74E+05
Salicyladehyde	90-02-8	68 h	69	16.82	3: 91 121 93	68		Medicinal		7.41E-03	1.33E+04	1.80E+06
Benzyl formate	104-57-4	68 h	70	17.06	4: 136 90 91 119	71		Floral			3.64E+04	

Compound	CAS	Ext. Time	Peak #	RT (min)	Models	Net % Match	Published Descriptors: Flavornet[1]	Published Descriptors: TGSC[2]	Published ODT (ppm): LRI & Odour Database[3]	Published ODT (ppm): Devos, et al. [4]	Surrogate Conc. (PAC)	OAV
2-methyl-1H-imidazole	693-98-1	68 h	71	17.13	4: 82 148 43 81	68					2.64E+04	
α-terpineol	98-55-5	1 h	23	17.74	TIC	80	Oil, Anise, Mint	Floral		3.72E-02	3.75E+04	1.01E+06
		68 h	72	17.74	17: 136 59 112 139 81 92 67 95 122 79 68 80 78 121 54 105 51	91	Oil, Anise, Mint	Floral		3.72E-02	4.43E+05	1.19E+07
Acetamide	60-35-5	68 h	73	17.88	1: 43	70		Mousy		6.03E+01	5.33E+04	8.85E+02
Benzyl acetate	140-11-4	68 h	74	18.05	5: 107 91 90 108 150	80	Fresh, Boiled vegetable	Sweet, Floral, Fruity, Jasmine, Fresh		1.45E-01	8.01E+04	5.54E+05
Verbenone	80-57-9	1 h	24	18.16	TIC	75		Camphor, Menthol, Celery			9.46E+03	
DL-carvone	99-49-0	68 h	75	18.71	4: 108 54 93 79	70	Mint, Basil, Fennel	Minty, Licorice		2.24E-02	6.11E+04	2.73E+06
Methyl acetylsalicylate	580-02-9	68 h	76	18.88	6: 121 152 153 64 65 43	93					2.13E+05	
Methyl salicylate	119-36-8	68 h	77	18.88	TIC	93	Peppermint	Minty		4.37E-02	1.31E+05	3.00E+06
(+)-sativene	3650-28-0	1 h	25	19.70	20: 93 69 120 148 106 68 55 92 189 95 149 175 135 162 190 136 83 91 53 103	75					2.37E+07	
Tyramine	51-67-2	1 h	27	19.73	6: 51 85 38 62 90 75	70		Meaty			9.20E+06	
β-caryophyllene	87-44-5	68 h	78	19.74	3: 122 56 110	83	Wood, Spice	Spice	6.40E-02		4.77E+06	7.46E+07
Benzyl Alcohol	100-51-6	5 min	25	19.75	7: 107 79 51 108 89 105 78	89	Sweet, Flower	Floral			2.31E+05	
		1 h	26	19.71	11: 78 53 109 149 39 129 66 65 123 134 202	96	Sweet, Flower	Floral			2.16E+07	
		68 h	79	19.77	TIC	100	Sweet, Flower	Floral			1.47E+08	

Compound	CAS	Ext. Time	Peak #	RT (min)	Models	Net % Match	Published Descriptors: Flavornet[1]	Published Descriptors: TGSC[2]	Published ODT (ppm): LRI & Odour Database[3]	Published ODT (ppm): Devos, et al. [4]	Surrogate Conc. (PAC)	OAV
α-terpinene	99-86-5	68 h	80	20.00	15: 105 80 94 136 106 68 91 161 40 65 189 43 55 135 83	70	Lemon	Woody			3.84E+05	
α-longipinene	5989-08-2	68 h	81	20.02	19: 121 136 119 161 122 91 55 81 41 67 135 39 189 78 80 53 137 56 82	76					6.13E+05	
Phenylethyl alcohol	60-12-8	68 h	82	20.38	3: 92 122 91	76	Honey, Spice, Rose, Lilac	Floral		1.70E-02	4.48E+04	2.64E+06
2-methyl naphthalene	91-57-6	68 h	83	20.44	7: 142 116 141 139 221 115 211	93		Floral			2.47E+05	
α-humulene	6753-98-6	1 h	28	20.55	20: 93 80 121 107 79 92 147 91 70 41 105 109 205 94 122 189 106 82 204 95	98	Wood	Wood	1.20E-01		3.99E+06	3.32E+07
		68 h	84	20.57	20: 93 121 80 67 92 189 147 94 204 91 53 41 107 95 106 79 55 161 109 77	97	Wood	Wood	1.20E-01		3.65E+06	3.04E+07
Benzyl nitrile	140-29-4	68 h	85	20.71	2: 90 116	71					8.20E+04	
α-cubebene	17699-14-8	68 h	86	20.92	4: 161 105 119 193	66	Herb, Wax	Herb			3.82E+04	
β-selinene	17066-67-0	1 h	29	21.28	17: 147 205 68 133 161 148 189 105 175 93 107 135 109 123 53 69 134	95	Herb	Herb			6.66E+05	

							Published Descriptors		Published ODT (ppm)			
**Compound**	**CAS**	**Ext. Time**	**Peak #**	**RT (min)**	**Models**	**Net % Match**	**Flavornet[1]**	**TGSC[2]**	**LRI & Odour Database[3]**	**Devos, et al. [4]**	**Surrogate Conc. (PAC)**	**OAV**
		68 h	87	21.29	20: 95 160 81 135 108 80 149 67 119 106 41 109 205 175 94 53 141 136 52 190	98	Herb	Herb			2.92E+06	
Aromadendrene	489-39-4	1 h	32	21.70	TIC	71	Wood	Wood			8.73E+04	
		68 h	88	21.30	20: 91 79 108 107 105 121 93 204 161 95 81 119 145 92 147 67 83 106 122 77	79	Wood	Wood			3.56E+06	
α-guaiene	3691-12-1	1 h	30	21.38	TIC	72	Wood, Balsamic	Wood			3.36E+05	
		68 h	92	21.72	20: 121 204 148 107 83 122 120 129 189 84 115 79 41 77 106 95 133 53 147 55	79	Wood, Balsamic	Wood			1.32E+06	
α-gurjunene	489-40-7	1 h	31	21.40	TIC	88	Wood, Balsamic	Wood			2.34E+05	
		68 h	89	21.44	20: 69 41 91 109 134 108 43 92 149 65 147 189 135 206 52 119 96 120 42 136	86	Wood, Balsamic	Wood			7.00E+06	
β-cedrene	546-28-1	68 h	90	21.45	11: 69 148 41 94 67 96 92 80 136 53 68	76					1.93E+06	
Phenol	108-95-2	68 h	91	21.64	TIC	90	Phenolic	Phenolic		1.10E-01	2.37E+05	2.16E+06
Dyclocaine	586-60-7	1 h	33	21.70	8: 120 105 67 109 122 121 91 119	66					1.69E+05	
		68 h	93	21.74	6: 83 145 123 104 95 159	67					2.71E+05	

							Published Descriptors		Published ODT (ppm)			
Compound	CAS	Ext. Time	Peak #	RT (min)	Models	Net % Match	Flavornet[1]	TGSC[2]	LRI & Odour Database[3]	Devos, et al. [4]	Surrogate Conc. (PAC)	OAV
(+)-calarene	17334-55-3	1 h	34	21.79	17: 147 109 161 91 148 204 135 133 92 189 107 94 93 159 134 41 149	78					6.42E+05	
		68 h	94	21.81	20: 161 204 147 162 108 133 106 105 91 107 65 176 67 95 160 135 150 189 109 41	84					2.84E+06	
α-cedrene	469-61-4	1 h	35	22.09	15: 119 204 161 93 65 69 133 80 121 135 134 41 189 94 79	72		Woody, Cedar, Sweet, Fresh			4.47E+05	
Valencene	4630-07-3	1 h	36	22.20	20: 161 204 131 133 91 53 106 190 68 108 43 66 77 94 162 78 148 73 160 143	96	Green, Oil	Citrus			3.46E+06	
		68 h	95	22.25	20: 161 133 135 91 93 119 51 107 67 81 77 104 41 55 63 175 108 136 94 132	96	Green, Oil	Citrus			8.12E+06	
γ-gurjunene	22567-17-5	1 h	37	22.30	20: 93 147 77 105 129 108 79 189 119 81 91 135 106 175 131 145 205 51 95 109	92		Musty			4.50E+06	
		68 h	96	22.34	20: 161 122 204 107 105 91 93 81 149 67 77 108 147 148 109 134 120 106 65 136	92		Musty			4.02E+07	

Compound	CAS	Ext. Time	Peak #	RT (min)	Models	Net % Match	Published Descriptors: Flavornet[1]	Published Descriptors: TGSC[2]	Published ODT (ppm): LRI & Odour Database[3]	Published ODT (ppm): Devos, et al. [4]	Surrogate Conc. (PAC)	OAV
Dimethyl-benzylcarbinyl acetate	151-05-3	68 h	97	22.43	TIC	72		Sweet, Floral, Fruity, Rose, Green, Pear, Berry, Jasmine, Powdery			1.42E+06	
2,6-dimethylquinoline	877-43-0	68 h	98	23.17	15: 157 153 200 218 164 158 156 115 63 68 105 91 128 201 139	74					3.28E+05	
2-phenoxyethanol	122-99-6	68 h	99	23.76	18: 94 138 77 79 39 119 95 91 45 51 232 50 78 92 182 96 75 125	94		Mild, Rose, Balsam, Cinnamyl			1.18E+06	
Nerolidol	7212-44-4	68 h	100	23.98	20: 69 41 81 43 79 163 108 121 123 136 97 91 178 120 119 94 140 160 133 155	78	Wood, Flower, Wax	Floral			2.92E+06	
(+)-nerolidol	142-50-7	68 h	101	23.99	20: 69 107 123 80 163 110 133 161 91 150 120 162 68 119 105 95 77 70 92 149	80		Floral			3.11E+06	
Caryophyllene oxide	1139-30-6	1 h	38	24.09	TIC	74	Herb, Sweet, Spice	Woody			2.69E+04	
		68 h	102	24.11	TIC	90	Herb, Sweet, Spice	Woody			1.99E+06	
Methyl anthranilate	134-20-3	68 h	103	25.23	5: 120 119 151 98 65	87	Honey, Flower	Fruity		1.15E-03	5.83E+04	5.07E+07
2,4-di-tert-butylphenol	96-76-4	68 h	104	26.37	3: 191 74 206	74		Phenolic			1.37E+05	
α-bisabolol	72691-24-8	1 h	39	26.43	TIC	77					3.95E+04	

Compound	CAS	Ext. Time	Peak #	RT (min)	Models	Net % Match	Published Descriptors: Flavornet[1]	Published Descriptors: TGSC[2]	Published ODT (ppm): LRI & Odour Database[3]	Published ODT (ppm): Devos, et al. [4]	Surrogate Conc. (PAC)	OAV
		68 h	105	26.45	20: 109 43 41 95 121 94 71 147 122 55 68 148 81 77 91 80 56 97 190 134	98					6.77E+06	
(-)-Globulol	489-41-8	68 h	106	27.33	8: 79 222 190 83 133 92 94 39	73					6.55E+05	
Diethyl Phthalate	84-66-2	68 h	107	27.45	12: 149 177 122 76 176 105 222 121 75 194 178 151	97					1.62E+06	
Benzophenone	119-61-9	68 h	108	28.79	TIC	85		Balsam, Rose, Metallic, Powdery, Geranium			7.99E+04	

A total of 124 chemical peaks were tentatively identified by *multidimensional* GC-MS. [1] Acree TE, Arn H. Flavornet and human odor space [Internet]. Geneva, NY: Cornell University; [2004; cited 2014 August 08]. Available from: http://flavornet.org/flavornet.html [2] The Good Scents Company Information System [Internet]. Oak Creek, WI; [1994; cited 2014 August 08]. Available from: http://www.thegoodscentscompany.com/index.html# [3] Mottram R. LRI & Odour Database [Internet]. UK: University of Reading; [2006; cited 2014 August 08]. Available from: www.odour.org.uk/index.html [4] Devos M, Patte F, Rouault J, Laffort P, Van Gemert LJ, editors. Standardized Human Olfactory Thresholds. IRL Press at Oxford Press; 1990. Peak # refers to chronological order compounds eluted from the analytical GC column; RT refers to retention time in min; Quantifying Ions are the component ions (number of ions used to quantify :corresponding m/z in order of relative abundance) or total ion chromatogram (TIC) used in AMDIS target library search and signal integration; % Match is net percent probability match of unknown spectra to database spectra, calculated by AMDIS; Surrogate Conc. is MS response to each separated compound, in peak area counts (PAC) (i.e. the integrated area under the curve of each chromatographic peak); OAV is the calculated Odor Activity Value (ratio of surrogate concentration to odor detection threshold), assuming equal mass detector response to all compounds, and assuming PAC units of ppm for illustrative purposes. All ODTs presented in this table are human olfactory thresholds in air.
