# Supplementary material for: Characterizing the Smell of Marijuana by Odor Impact of Volatile Compounds: An Application of Simultaneous Chemical and Sensory Analysis
Source: PLoS One. 2015 Dec 10;10(12):e0144160. doi: 10.1371/journal.pone.0144160 (PMC4684335; doi:10.1371/journal.pone.0144160)
Supplement: S3 Table — (PDF) [file pone.0144160.s008.pdf]

Table S3. Summary of VOC emitted from unpackaged marijuana into headspace and captured by SPME during 5 min, 1 h, 68 h static sampling at room temperature.

							Published Descriptors		Published ODT (ppm)			
**Compound**	**CAS**	**Ext. Time**	**Peak #**	**RT (min)**	**Models**	**Net % Match**	**Flavornet[1]**	**TGSC[2]**	**LRI & Odour Database[3]**	**Devos, et al. [4]**	**Surrogate Conc. (PAC)**	**OAV**
Ethylene oxide	75-21-8	68 h	1	1.06	2: 44 46	66				8.51E+02	1.13E+06	1.33E+03
Butyl formate	592-84-7	1 h	1	1.13	3: 41 39 56	65		Fruity			2.32E+04	
		68 h	2	1.13	2: 39 56	67		Fruity			8.34E+03	
Acetaldehyde	75-07-0	1 h	2	1.20	2: 43 42	88	Pungent, Ether	Pungent, Ethereal, Aldehydic, Fruity		1.86E-01	8.62E+03	4.63E+04
Isobutane	75-28-5	5 min	1	1.23	12: 43 42 41 57 72 39 50 55 58 54 56 37	84				1.00E+01	1.21E+06	1.21E+05
		68 h	3	1.24	9: 43 42 41 56 71 53 61 37 58	85				1.00E+01	2.27E+06	2.27E+05
		1 h	3	1.24	10: 43 42 41 57 39 72 55 56 73 37	85				1.00E+01	2.94E+06	2.94E+05
Methyl mercaptan	74-93-1	68 h	4	1.27	3: 47 48 45	84	Sulfur, Gasoline, Garlic	Decomposing, Cabbage, Garlic			1.13E+04	
Isoprene	78-79-5	68 h	5	1.33	3: 67 68 39	86					5.78E+04	
		1 h	4	1.34	TIC	93					3.12E+04	
2-methylpentane	107-83-5	5 min	2	1.39	3: 71 43 42	97					2.82E+05	
		1 h	5	1.40	12: 42 41 55 39 69 72 70 86 56 40 65 50	97					6.18E+05	
		68 h	6	1.40	10: 43 41 71 70 42 57 55 39 86 56	97					3.99E+05	
3,4,5-trimethyl-1-hexene	56728-10-0	5 min	3	1.40	4: 41 86 39 70	68					2.59E+05	
		1 h	6	1.40	TIC	67					6.20E+05	
2,3,4-trimethylpentane	565-75-3	1 h	7	1.40	9: 43 70 41 55 57 53 56 54 50	77					2.03E+05	
3-methylpentane	96-14-0	5 min	4	1.45	2: 56 57	86					7.49E+04	
		68 h	7	1.45	TIC	93					5.09E+04	

							Published Descriptors		Published ODT (ppm)			
Compound	CAS	Ext. Time	Peak #	RT (min)	Models	Net % Match	Flavornet[1]	TGSC[2]	LRI & Odour Database[3]	Devos, et al. [4]	Surrogate Conc. (PAC)	OAV
		1 h	8	1.46	TIC	97					1.22E+05	
2-methylaziridine	75-55-8	1 h	9	1.46	5: 57 56 41 53 39	81					1.67E+05	
Dimethylsulfide	75-18-3	1 h	10	1.51	5: 46 45 47 61 35	94	Cabbage, Sulfur, Gasoline	Sulfury, Onion, Sweet corn, Vegetable, Cabbage, Tomato, Green, Radish		2.24E-03	9.43E+04	4.21E+07
		68 h	8	1.51	1: 47	88	Cabbage, Sulfur, Gasoline	Sulfury, Onion, Sweet corn, Vegetable, Cabbage, Tomato, Green, Radish		2.24E-03	4.04E+04	1.80E+07
Propanal	123-38-6	1 h	11	1.57	TIC	76	Solvent, Pungent	Earthy, Alcohol, Wine, Whiskey, Cocoa, Nutty		2.69E-02	3.30E+04	1.23E+06
		68 h	9	1.57	1: 58	75	Solvent, Pungent	Earthy, Alcohol, Wine, Whiskey, Cocoa, Nutty		2.69E-02	4.70E+04	1.75E+06
Butane	106-97-8	1 h	12	1.65	5: 41 59 44 37 60	84				2.04E+02	1.88E+06	9.21E+03
Acetone	67-64-1	5 min	5	1.65	TIC	83		Solvent		1.45E+01	7.16E+04	4.95E+03
		1 h	13	1.66	10: 43 58 42 39 41 38 37 44 36 59	99		Solvent		1.45E+01	4.98E+06	3.45E+05
		68 h	10	1.65	6: 43 58 42 39 41 44	99		Solvent		1.45E+01	1.97E+06	1.36E+05
Isobutyraldehyde	78-84-2	1 h	14	1.77	TIC	88	Pungent, Malt, Green	Spicy		4.07E-02	1.52E+04	3.73E+05

							Published Descriptors		Published ODT (ppm)			
**Compound**	**CAS**	**Ext. Time**	**Peak #**	**RT (min)**	**Models**	**Net % Match**	**Flavornet[1]**	**TGSC[2]**	**LRI & Odour Database[3]**	**Devos, et al. [4]**	**Surrogate Conc. (PAC)**	**OAV**
Methacrolein	78-85-3	1 h	15	2.14	TIC	87		Wild hyacinth foliage			3.42E+04	
		68 h	11	2.13	TIC	96		Wild hyacinth foliage			1.13E+05	
Ethanol	64-17-5	5 min	6	2.33	2: 45 46	95	Sweet	Alcoholic		2.88E+01	1.70E+05	5.90E+03
		1 h	16	2.33	6: 45 46 39 42 41 47	99	Sweet	Alcoholic		2.88E+01	3.01E+05	1.04E+04
		68 h	12	2.34	2: 45 42	91	Sweet	Alcoholic		2.88E+01	3.02E+05	1.05E+04
Methylene chloride	75-09-2	1 h	17	2.42	5: 84 39 86 88 47	95				2.82E+01	7.08E+04	2.51E+03
Pentanal	110-62-3	68 h	13	3.66	TIC	68	Almond, Malt, Pungent	Fermented		6.03E-03	9.80E+03	1.63E+06
tert-butanol	75-65-0	5 min	8	3.94	TIC	75		Camphor			3.37E+04	
		68 h	14	3.92	5: 33 43 59 40 57	77		Camphor			4.49E+05	
3-pentanol	584-02-1	1 h	19	3.92	TIC	71	Fruit	Herbal		4.68E-01	1.21E+05	2.58E+05
Ethylenediamine	107-15-3	5 min	7	3.93	TIC	76					1.94E+05	
1,1-dimethyl-hydrazine	57-14-7	5 min	9	3.94	TIC	79					9.60E+04	
		1 h	18	3.91	TIC	83					1.41E+05	
		68 h	15	3.93	3: 60 58 45	91					1.01E+05	
Hydrazine	302-01-2	5 min	10	3.96	2: 33 61	77		Ammoniacal		3.00E+00	3.41E+03	1.14E+03
		1 h	20	3.92	3: 33 45 37	77				3.00E+00	4.44E+03	1.48E+03
		68 h	16	3.93	2: 33 61	77				3.00E+00	1.03E+04	3.42E+03
Hexanal	66-25-1	1 h	21	5.99	TIC	84	Grass, Tallow, Fat	Green		1.38E-02	3.44E+04	2.49E+06
		68 h	17	5.98	9: 41 43 56 44 55 57 40 50 54	96	Grass, Tallow, Fat	Green		1.38E-02	7.43E+05	5.39E+07
1-butanol	71-36-3	68 h	18	6.12	TIC	91	Medicine, Fruit	Fermented		4.90E-01	4.11E+04	8.39E+04
Nonane	111-84-2	68 h	19	6.68	TIC	87	Alkane	Gasoline		1.26E+00	4.14E+04	3.29E+04
2-isopropenyl-3-methylpyrazine	145984-65-2	1 h	22	7.67	4: 135 75 134 133	73					1.03E+05	
α-phellandrene	99-83-2	5 min	12	7.89	TIC	82	Turpentine, Mint, Spice	Terpenic			2.36E+04	
		1 h	25	7.90	11: 92 136 91 93 108 78 39 77 107 106 66	79	Turpentine, Mint, Spice	Terpenic			1.55E+06	

Compound	CAS	Ext. Time	Peak #	RT (min)	Models	Net % Match	Published Descriptors: Flavornet[1]	Published Descriptors: TGSC[2]	Published ODT (ppm): LRI & Odour Database[3]	Published ODT (ppm): Devos, et al. [4]	Surrogate Conc. (PAC)	OAV
(+)-4-Carene	29050-33-7	1 h	23	7.90	13: 121 78 136 68 103 117 80 52 51 77 106 107 81	82			4.00E+00		2.20E+05	5.49E+04
Sabinene	3387-41-5	1 h	26	7.92	7: 91 67 107 108 41 94 63	69	Pepper, Turpentine, Wood	Woody			1.09E+05	
γ-terpinene	99-85-4	1 h	27	7.92	7: 77 107 80 121 92 137 63	69	Gasoline, Turpentine	Terpenic			1.64E+05	
α-pinene	80-56-8	5 min	11	7.89	TIC	79	Pine, Turpentine	Herbal		6.92E-01	2.36E+04	3.41E+04
		1 h	24	7.90	11: 92 136 91 93 108 78 39 77 107 106 66	97	Pine, Turpentine	Herbal		6.92E-01	4.88E+05	7.05E+05
		68 h	20	7.92	7: 80 91 121 92 79 105 107	93	Pine, Turpentine	Herbal		6.92E-01	1.88E+05	2.71E+05
2-heptanone	110-43-0	68 h	21	8.44	TIC	90	Soap	Cheesy		1.41E-01	2.35E+05	1.66E+06
Heptanal	111-71-7	68 h	22	8.61	TIC	97	Fat, Citrus, Rancid	Green		4.79E-03	1.90E+06	3.97E+08
4-methylpyrimidine	3438-46-8	68 h	23	9.05	TIC	90					7.71E+04	
4-pyridinamine	504-24-5	68 h	24	9.05	4: 67 52 53 41	86					1.03E+05	
1,3,5-triazine-2,4,6-triamine	108-78-1	5 min	13	9.26	2: 126 84	68					4.66E+03	
Styrene	100-42-5	68 h	25	9.42	3: 104 102 50	92	Balsamic, Gasoline	Balsamic		1.45E-01	1.32E+05	9.11E+05
Myrcene	123-35-3	1 h	28	9.92	TIC	94	Balsamic, Must, Spice	Peppery, Terpene, Spicy, Balsam, Plastic	1.30E-02		6.11E+05	4.70E+07
		68 h	26	9.93	9: 137 50 117 103 74 89 51 58 138	96	Balsamic, Must, Spice	Peppery, Terpene, Spicy, Balsam, Plastic	1.30E-02		9.74E+06	7.49E+08
β-pinene	18172-67-3	68 h	27	9.94	TIC	97	Pine, Resin, Turpentine	Terpenic			1.62E+07	

Compound	CAS	Ext. Time	Peak #	RT (min)	Models	Net % Match	Published Descriptors		Published ODT (ppm)		Surrogate Conc. (PAC)	OAV
							Flavornet[1]	TGSC[2]	LRI & Odour Database[3]	Devos, et al. [4]		
Dimethylpyrazine	123-32-0	68 h	28	10.61	TIC	70	Cocoa, Roasted nut, Roast beef, Medicine	Cocoa, Roasted nuts, Roast Beef, Woody, Grass, Medical			6.43E+03	
1-hexanol	111-27-3	68 h	29	10.73	4: 42 56 41 84	80	Resin, Flower, Breen	Herbal		4.37E-02	2.08E+05	4.75E+06
Diacetone alcohol	123-42-2	1 h	29	10.80	2: 59 43	77				8.91E-01	5.51E+04	6.18E+04
		68 h	30	10.80	1: 101	71				8.91E-01	8.98E+04	1.01E+05
Camphene	79-92-5	1 h	30	10.89	7: 79 68 136 107 92 95 91	84	Camphor	Woody			1.33E+05	
		68 h	32	10.89	TIC	89	Camphor	Woody			1.23E+06	
Limonene	138-86-3	1 h	31	10.90	6: 68 92 80 136 69 41	90	Lemon, Orange	Citrus		4.37E-01	1.71E+05	3.92E+05
		68 h	31	10.89	TIC	95	Lemon, Orange	Citrus		4.37E-01	1.23E+06	2.83E+06
Octanal	124-13-0	68 h	33	11.20	TIC	85	Fat, Soap, Lemon, Green	Aldehydic, Waxy, Citrus, Orange peel, Green, Fatty		1.35E-03	1.07E+05	7.96E+07
m-cymene	535-77-3	1 h	32	11.36	TIC	86					3.49E+04	
		68 h	34	11.37	TIC	92					1.22E+05	
Methylisohexenyl ketone	110-93-0	1 h	33	11.54	TIC	80	Pepper, Mushroom, Rubber	Citrus		3.80E-02	4.51E+04	1.19E+06
		68 h	35	11.52	13: 93 58 67 111 56 71 42 53 38 82 54 44 70	97	Pepper, Mushroom, Rubber	Citrus		3.80E-02	1.48E+06	3.90E+07
δ-3-carene	13466-78-9	68 h	36	11.62	TIC	97	Lemon, Resin	Citrus	4.00E+00		1.49E+06	3.72E+05
2-butoxyethanol	111-76-2	68 h	37	11.75	7: 45 87 41 42 75 39 88	89				3.39E-01	1.41E+05	4.16E+05
Undecane	1120-21-4	68 h	38	11.87	TIC	92	Alkane			1.17E+00	1.31E+05	1.12E+05
Acetic acid	64-19-7	1 h	34	12.25	3: 43 44 207	100	Sour	Acidic		1.45E-01	7.66E+05	5.30E+06
		68 h	39	12.21	7: 43 45 60 44 40 207 59	99	Sour	Acidic		1.45E-01	6.49E+06	4.49E+07

Compound	CAS	Ext. Time	Peak #	RT (min)	Models	Net % Match	Published Descriptors: Flavornet[1]	Published Descriptors: TGSC[2]	Published ODT (ppm): LRI & Odour Database[3]	Published ODT (ppm): Devos, et al. [4]	Surrogate Conc. (PAC)	OAV
Furfural	98-01-1	68 h	40	12.68	2: 96 39	96	Bread, Almond, Sweet	Sweet, Woody, Almond, Baked bread		7.76E-01	1.64E+05	2.11E+05
Citronellolformate	105-85-1	68 h	41	13.08	7: 120 41 92 96 93 109 138	71		Floral			4.20E+05	
1,3-dichlorobenzene	541-73-1	68 h	42	13.15	6: 75 111 84 76 150 147	97					3.07E+05	
2-ethylhexanol	104-76-7	1 h	35	13.80	7: 84 41 54 112 43 56 70	91	Rose, Green	Citrus		2.45E-01	3.39E+05	1.38E+06
		68 h	43	13.83	TIC	92	Rose, Green	Citrus		2.45E-01	1.28E+06	5.22E+06
2-ethenyl-1,3-dimethylbenzene	2039-90-9	68 h	44	13.93	4: 104 89 117 115	81					6.28E+04	
Benzaldehyde	100-52-7	5 min	15	14.11	2: 106 77	87	Almond, Burnt sugar	Fruity		4.17E-02	4.75E+04	1.14E+06
		1 h	36	14.06	TIC	98	Almond, Burnt sugar	Fruity		4.17E-02	1.00E+06	2.40E+07
		68 h	45	14.05	14: 106 105 51 78 107 52 74 39 75 79 63 37 108 36	99	Almond, Burnt sugar	Fruity		4.17E-02	3.24E+07	7.76E+08
2-chloroacetophenone	532-27-4	5 min	14	14.09	2: 105 52	68				2.57E-02	2.00E+04	7.80E+05
Dodecane	112-40-3	68 h	46	14.19	8: 170 98 84 85 127 147 58 269	68	Alkane	Alkane		2.04E+00	4.12E+05	2.02E+05
1-(3-methylphenyl)-ethanone	585-74-0	68 h	47	14.46	TIC	85					3.97E+04	
tert-butyl-benzene	98-06-6	68 h	48	14.48	8: 79 117 119 78 134 135 92 120	87					1.37E+05	
Linalool	78-70-6	1 h	37	15.12	20: 93 69 80 71 72 122 41 92 55 107 136 65 94 53 81 105 45 56 96 82	95	Flower, Lavender	Floral		5.37E-02	3.68E+05	6.85E+06

							Published Descriptors		Published ODT (ppm)			
Compound	CAS	Ext. Time	Peak #	RT (min)	Models	Net % Match	Flavornet[1]	TGSC[2]	LRI & Odour Database[3]	Devos, et al. [4]	Surrogate Conc. (PAC)	OAV
		68 h	50	15.13	20: 41 43 69 121 80 106 67 83 92 39 94 79 72 97 68 136 57 96 82 107	98	Flower, Lavender	Floral		5.37E-02	3.65E+06	6.79E+07
Isobornyl thiocyanoacetate	115-31-1	68 h	49	15.12	15: 39 96 109 154 42 56 84 95 65 85 139 58 54 44 57	73					2.51E+06	
cis-2-pinanol	4948-29-2	1 h	38	15.41	TIC	79		Herbal			1.66E+04	
Benzonitrile	100-47-0	68 h	51	15.47	2: 103 91	80	Rancid, Sweet				1.56E+05	
α-ionol	25312-34-9	68 h	52	15.66	19: 138 95 67 79 43 39 86 77 123 42 96 91 139 41 93 71 55 137 44	80		Ionone, Tropical, Sweet, Floral, Violet, Woody			1.08E+06	
Fenchyl alcohol	1632-73-1	1 h	39	15.73	TIC	72	Camphor	Camphor, Borneol, Pine, Woody, Dry, Sweet, Lemon			1.94E+04	
2-ethoxyethanol	110-80-5	68 h	53	15.79	3: 104 59 72	67				1.23E+00	1.06E+05	8.63E+04
Decanal	112-31-2	68 h	54	15.89	TIC	84	Soap, Orange peel, Tallow	Aldehydic		8.91E-04	1.45E+05	1.63E+08
Methyl heptadienone	1604-28-0	68 h	55	15.91	TIC	86		Cinnamon, Coconut, Spice, Woody, Sweet, Weedy			7.94E+04	
Methyl benzoate	93-58-3	68 h	56	16.25	1: 105	82	Prune, Lettuce, Herb, Sweet	Phenolic		1.07E-01	3.90E+04	3.64E+05
Tridecane	629-50-5	68 h	57	16.35	4: 70 84 73 56	82	Alkane	Alkane		2.14E+00	1.38E+05	6.47E+04
Acetophenone	98-86-2	68 h	58	16.51	3: 105 90 106	87	Musty, Flower, Almond	Floral		3.63E-01	7.91E+04	2.18E+05
Salicyladehyde	90-02-8	68 h	59	16.81	TIC	67		Medicinal		7.41E-03	2.70E+04	3.64E+06
Benzyl formate	104-57-4	68 h	60	17.04	6: 91 136 90 89 78 51	92		Floral			1.69E+05	

Compound	CAS	Ext. Time	Peak #	RT (min)	Models	Net % Match	Published Descriptors: Flavornet[1]	Published Descriptors: TGSC[2]	Published ODT (ppm): LRI & Odour Database[3]	Published ODT (ppm): Devos, et al. [4]	Surrogate Conc. (PAC)	OAV
2-methyl-1H-imidazole	693-98-1	68 h	61	17.12	3: 82 97 54	82					3.89E+04	
α-terpineol	98-55-5	1 h	40	17.73	TIC	80	Oil, Anise, Mint	Floral		3.72E-02	2.54E+04	6.84E+05
		68 h	62	17.73	8: 121 68 81 136 107 77 109 69	93	Oil, Anise, Mint	Floral		3.72E-02	9.94E+05	2.68E+07
Acetamide	60-35-5	68 h	63	17.89	10: 146 69 128 117 120 45 115 105 161 134	87		Mousy		6.03E+01	3.78E+05	6.28E+03
Benzyl acetate	140-11-4	68 h	64	18.03	9: 150 109 108 91 79 107 51 50 83	94	Fresh, Boiled vegetable	Sweet, Floral, Fruity, Jasmine, Fresh		1.45E-01	2.68E+05	1.85E+06
m-tert-butylphenol	585-34-2	1 h	41	18.15	5: 135 80 108 79 91	68					6.76E+04	
		68 h	65	18.15	8: 108 107 79 91 150 115 39 110	69					2.05E+04	
p-tert-butylphenol	98-54-4	1 h	42	18.15	TIC	68		Leathery			1.54E+04	
Verbenone	80-57-9	1 h	43	18.16	TIC	82		Camphor, Menthol, Celery			2.55E+04	
		68 h	66	18.16	8: 107 91 149 55 146 150 73 108	68		Camphor, Menthol, Celery			5.53E+04	
DL-carvone	99-49-0	68 h	67	18.68	4: 107 82 54 93	82	Mint, Basil, Fennel	Minty, Licorice		2.24E-02	1.56E+05	6.97E+06
Methyl acetylsalicylate	580-02-9	68 h	68	18.86	4: 82 125 120 43	90					5.82E+04	
Methyl salicylate	119-36-8	68 h	69	18.87	5: 120 152 121 43 63	88	Peppermint	Minty		4.37E-02	1.09E+05	2.50E+06
β-caryophyllene	87-44-5	5 min	16	19.67	TIC	92	Wood, Spice	Spice	6.40E-02		3.14E+05	4.91E+06
		1 h	44	19.65	10: 134 124 96 66 112 190 122 110 177 138	89	Wood, Spice	Spice	6.40E-02		1.05E+06	1.64E+07

							Published Descriptors		Published ODT (ppm)			
**Compound**	**CAS**	**Ext. Time**	**Peak #**	**RT (min)**	**Models**	**Net % Match**	**Flavornet[1]**	**TGSC[2]**	**LRI & Odour Database[3]**	**Devos, et al. [4]**	**Surrogate Conc. (PAC)**	**OAV**
		68 h	70	19.69	20: 133 93 69 120 41 147 148 67 189 81 94 95 175 82 135 162 137 129 122 136	80	Wood, Spice	Spice	6.40E-02		5.25E+06	8.20E+07
(+)-sativene	3650-28-0	68 h	71	19.72	3: 105 86 37	73					1.40E+07	
Tyramine	51-67-2	5 min	18	19.74	3: 80 51 49	70		Meaty			6.64E+05	
		1 h	46	19.73	5: 90 62 109 37 61	70		Meaty			3.71E+06	
Benzyl Alcohol	100-51-6	5 min	17	19.73	5: 90 80 91 51 74	97	Sweet, Flower	Floral			5.67E+05	
		1 h	45	19.72	TIC	96	Sweet, Flower	Floral			5.83E+06	
		68 h	72	19.79	11: 79 107 51 91 63 109 74 49 40 48 155	100	Sweet, Flower	Floral			1.86E+08	
α-terpinene	99-86-5	68 h	73	19.98	15: 121 109 122 136 161 162 123 41 120 107 81 91 190 204 92	67	Lemon	Woody			2.09E+05	
α-longipinene	5989-08-2	68 h	74	20.01	4: 119 109 91 40	73					4.73E+05	
Dimethylsulfone	67-71-0	1 h	47	20.11	2: 94 79	80	Sulfur, Burnt	Sulfurous, Burnt			1.93E+04	
		68 h	75	20.10	7: 94 81 82 54 119 46 150	82	Sulfur, Burnt	Sulfurous, Burnt			1.54E+05	
Phenylethyl alcohol	60-12-8	68 h	76	20.39	5: 91 122 92 44 57	82	Honey, Spice, Rose, Lilac	Floral		1.70E-02	4.72E+04	2.78E+06
2-methyl naphthalene	91-57-6	68 h	77	20.43	5: 141 139 115 205 147	81		Floral			6.87E+04	
α-humulene	6753-98-6	5 min	19	20.53	TIC	83	Wood	Wood	1.20E-01		4.30E+04	3.58E+05
		1 h	48	20.52	19: 92 79 94 105 95 91 148 63 65 204 120 41 123 82 135 78 66 39 128	97	Wood	Wood	1.20E-01		1.42E+06	1.19E+07

							Published Descriptors		Published ODT (ppm)			
**Compound**	**CAS**	**Ext. Time**	**Peak #**	**RT (min)**	**Models**	**Net % Match**	**Flavornet[1]**	**TGSC[2]**	**LRI & Odour Database[3]**	**Devos, et al. [4]**	**Surrogate Conc. (PAC)**	**OAV**
		68 h	78	20.56	20: 136 106 94 119 204 55 190 82 115 95 161 54 189 149 148 52 71 128 65 66	97	Wood	Wood	1.20E-01		5.44E+06	4.53E+07
Benzyl nitrile	140-29-4	68 h	79	20.70	5: 51 117 112 118 77	88					1.13E+05	
α-cubebene	17699-14-8	68 h	80	20.90	TIC	71	Herb, Wax	Herb			2.41E+04	
β-selinene	17066-67-0	1 h	50	21.25	15: 161 162 134 94 190 43 91 81 204 121 123 95 92 131 175	72	Herb	Herb			1.56E+05	
		68 h	82	21.26	20: 55 79 91 147 96 120 176 121 135 105 106 175 145 190 81 94 104 40 109 148	98	Herb	Herb			3.65E+06	
α-gurjunene	489-40-7	1 h	51	21.38	10: 147 131 107 133 109 204 119 79 95 105	81	Wood, Balsamic	Wood			1.34E+05	
		68 h	81	21.11	13: 204 93 161 127 189 133 95 122 169 128 190 117 123	89	Wood, Balsamic	Wood			4.08E+05	
β-cedrene	546-28-1	68 h	83	21.41	20: 161 93 134 107 77 122 94 41 121 120 67 106 95 66 54 39 135 163 108 119	76					1.62E+06	
Phenol	108-95-2	68 h	84	21.63	17: 94 66 65 47 62 95 63 64 40 38 74 90 55 61 49 53 36	93	Phenolic	Phenolic		1.10E-01	2.08E+06	1.89E+07
Aromadendrene	489-39-4	1 h	49	21.25	TIC	91	Wood	Wood			1.16E+05	

Compound	CAS	Ext. Time	Peak #	RT (min)	Models	Net % Match	Published Descriptors: Flavornet[1]	Published Descriptors: TGSC[2]	Published ODT (ppm): LRI & Odour Database[3]	Published ODT (ppm): Devos, et al. [4]	Surrogate Conc. (PAC)	OAV
		68 h	85	21.70	19: 121 204 189 83 120 105 148 122 147 91 79 93 107 119 95 134 157 54 103	77	Wood	Wood			1.81E+06	
α-guaiene	3691-12-1	68 h	86	21.71	20: 133 120 91 106 105 119 78 148 204 189 93 83 161 107 123 145 67 80 81 53	78	Wood, Balsamic	Wood			7.32E+05	
α-cedrene	469-61-4	1 h	52	22.07	TIC	72		Woody, Cedar, Sweet, Fresh			1.74E+04	
		68 h	87	22.10	20: 93 119 79 204 107 94 95 77 136 69 81 189 120 39 205 122 106 161 148 133	74		Woody, Cedar, Sweet, Fresh			6.98E+06	
Valencene	4630-07-3	5 min	20	22.18	TIC	76	Green, Oil	Citrus			1.85E+04	
		1 h	53	22.19	TIC	95	Green, Oil	Citrus			8.47E+05	
		68 h	88	22.21	19: 133 67 205 77 55 162 190 189 175 130 109 174 92 121 117 106 94 108 80	95	Green, Oil	Citrus			1.39E+07	
γ-gurjunene	22567-17-5	1 h	54	22.27	TIC	89		Musty			5.08E+05	
		68 h	89	22.32	20: 122 105 91 121 81 41 149 55 123 190 65 175 129 104 103 73 51 173 163 150	92		Musty			4.82E+07	

							Published Descriptors		Published ODT (ppm)			
**Compound**	**CAS**	**Ext. Time**	**Peak #**	**RT (min)**	**Models**	**Net % Match**	**Flavornet[1]**	**TGSC[2]**	**LRI & Odour Database[3]**	**Devos, et al. [4]**	**Surrogate Conc. (PAC)**	**OAV**
Dimethylbenzylcarbinyl acetate	151-05-3	68 h	90	22.41	6: 71 132 117 60 59 115	65		Sweet, Floral, Fruity, Rose, Green, Pear, Berry, Jasmine, Powdery			8.36E+05	
2,6-dimethylquinoline	877-43-0	68 h	91	23.13	15: 157 142 127 136 156 200 155 135 158 115 152 128 129 153 126	70					5.20E+05	
2-phenoxyethanol	122-99-6	68 h	92	23.77	6: 94 77 66 147 65 71	90		Mild, Rose, Balsam, Cinnamyl			1.63E+05	
Nerolidol	7212-44-4	68 h	93	23.96	20: 41 119 81 136 71 95 121 67 79 105 123 91 135 80 108 163 190 109 53 124	83	Wood, Flower, Wax	Floral			3.53E+06	
(+)-nerolidol	142-50-7	68 h	94	23.97	20: 69 93 107 81 202 55 79 121 109 161 123 67 133 53 178 77 137 163 204 97	86		Floral			3.42E+06	
Caryophyllene oxide	1139-30-6	68 h	95	24.09	20: 95 82 83 69 123 66 124 105 159 71 161 189 138 111 160 191 97 112 162 54	91	Herb, Sweet, Spice	Woody			3.19E+06	
Methyl anthranilate	134-20-3	68 h	96	25.24	8: 93 80 92 151 65 120 98 95	86	Honey, Flower	Fruity		1.15E-03	8.93E+04	7.78E+07
2,4-di-tert-butylphenol	96-76-4	68 h	97	26.36	4: 191 200 116 206	74		Phenolic			7.61E+04	

Compound	CAS	Ext. Time	Peak #	RT (min)	Models	Net % Match	Published Descriptors: Flavornet[1]	Published Descriptors: TGSC[2]	Published ODT (ppm): LRI & Odour Database[3]	Published ODT (ppm): Devos, et al. [4]	Surrogate Conc. (PAC)	OAV
α-bisabolol	72691-24-8	68 h	98	26.42	20: 204 95 110 67 122 92 139 84 81 148 161 97 137 133 68 123 78 83 140 190	98					7.43E+06	
(-)-Globulol	489-41-8	68 h	99	27.32	18: 81 121 151 119 95 123 204 133 91 148 222 189 145 55 149 41 79 82	73					8.23E+05	
Diethyl Phthalate	84-66-2	68 h	100	27.43	7: 149 65 222 50 119 150 93	93					4.00E+05	
Benzophenone	119-61-9	68 h	101	28.78	2: 105 93	80		Balsam, Rose, Metallic, Powdery, Geranium			9.39E+04	

A total of 121 chemical peaks were tentatively identified by *multidimensional* GC-MS; [1] Acree TE, Arn H. Flavornet and human odor space [Internet]. Geneva, NY: Cornell University; [2004; cited 2014 August 08]. Available from: http://flavornet.org/flavornet.html [2] The Good Scents Company Information System [Internet]. Oak Creek, WI; [1994; cited 2014 August 08]. Available from: http://www.thegoodscentscompany.com/index.html# [3] Mottram R. LRI & Odour Database [Internet]. UK: University of Reading; [2006; cited 2014 August 08]. Available from: www.odour.org.uk/index.html [4] Devos M, Patte F, Rouault J, Laffort P, Van Gemert LJ, editors. Standardized Human Olfactory Thresholds. IRL Press at Oxford Press; 1990. Peak # refers to chronological order compounds eluted from the analytical GC column; RT refers to retention time in min; Quantifying Ions are the component ions (number of ions used to quantify :corresponding m/z in order of relative abundance) or total ion chromatogram (TIC) used in AMDIS target library search and signal integration; % Match is net percent probability match of unknown spectra to database spectra, calculated by AMDIS; Surrogate Conc. is MS response to each separated compound, in peak area counts (PAC) (i.e. the integrated area under the curve of each chromatographic peak); OAV is the calculated Odor Activity Value (ratio of surrogate concentration to odor detection threshold), assuming equal mass detector response to all compounds, and assuming PAC units of ppm for illustrative purposes. All ODTs presented in this table are human olfactory thresholds in air.
