# Supplementary material for: Characterizing the Smell of Marijuana by Odor Impact of Volatile Compounds: An Application of Simultaneous Chemical and Sensory Analysis
Source: PLoS One. 2015 Dec 10;10(12):e0144160. doi: 10.1371/journal.pone.0144160 (PMC4684335; doi:10.1371/journal.pone.0144160)
Supplement: S4 Table — (PDF) [file pone.0144160.s009.pdf]

Table S4. Summary of F-statistics and p-values from two-way analysis of variance comparing the effect of packaging on VOC emitted from marijuana at 5 min, 1 h, and 68 h extraction times.

				Packaging		Ext. Time	
	R^2	F-Statistic	p-value	F-Statistic	p-value	F-Statistic	p-value
(-)-Globulol	0.987	51.765	0.019	1.000	0.423	77.148	0.013†
(+)-4-Carene	0.817	2.980	0.261	1.000	0.423	3.970	0.201
(+)-calarene	0.657	1.275	0.468	1.824	0.309	1.000	0.500
(+)-nerolidol	0.998	310.437	0.003	1.000	0.423	465.156	0.002†
(+)-sativene	0.302	0.288	0.834	0.085	0.798	0.390	0.719
1-(3-methylphenyl)-ethanone	0.600	1.000	0.535	1.000	0.423	1.000	0.500
1,1-dimethyl-hydrazine	0.423	0.489	0.725	0.012	0.922	0.728	0.579
1,3,5-triazine-2,4,6-triamine	0.914	7.097	0.126	1.000	0.423	10.145	0.090
1,3-dichlorobenzene	0.957	14.855	0.064	1.000	0.423	21.782	0.044◊
1-butanol	0.979	30.556	0.032	1.000	0.423	45.335	0.022†
1-butoxy-2-propanol	0.600	1.000	0.535	1.000	0.423	1.000	0.500
1-hexanol	0.961	16.559	0.057	1.000	0.423	24.338	0.039†
1-Propanamine, 3-dibenzo[b,e]thiepin-11(6H)-ylidene-N,N-dimethyl-, S-oxide	0.600	1.000	0.535	1.000	0.423	1.000	0.500
2,3,4-trimethylpentane	0.254	0.227	0.872	0.006	0.943	0.338	0.748
2,4-di-tert-butylphenol	0.927	8.506	0.107	1.000	0.423	12.259	0.075
2,6-dimethylquinoline	0.952	13.284	0.071	1.000	0.423	19.425	0.049◊
2-butoxyethanol	0.834	3.353	0.238	1.000	0.423	4.529	0.181
2-chloroacetophenone	0.712	1.646	0.400	3.183	0.216	0.878	0.533
2-ethenyl-1,3-dimethylbenzene	0.766	2.180	0.330	1.000	0.423	2.771	0.265
2-ethoxyethanol	0.793	2.554	0.294	1.000	0.423	3.331	0.231
2-ethylhexanol	0.962	16.695	0.057	2.675	0.244	23.706	0.040‡
2-heptanone	0.998	295.779	0.003	1.000	0.423	443.169	0.002†
2-isopropenyl-3-methylpyrazine	0.558	0.842	0.583	0.796	0.466	0.864	0.536
2-methyl naphthalene	0.785	2.428	0.305	1.000	0.423	3.142	0.241
2-methyl-1H-imidazole	0.965	18.596	0.051	1.000	0.423	27.395	0.035†
2-methylaziridine	0.303	0.289	0.833	0.155	0.732	0.357	0.737
2-methylpentane	0.730	1.803	0.376	0.753	0.477	2.328	0.300
2-nitropropane	0.715	1.676	0.395	3.028	0.224	1.000	0.500
2-phenoxyethanol	0.692	1.497	0.424	1.000	0.423	1.746	0.364
3,4,5-trimethyl-1-hexene	0.699	1.552	0.415	2.655	0.245	1.000	0.500
3-methylpentane	0.712	1.652	0.399	0.985	0.426	1.986	0.335
3-pentanol	0.600	1.000	0.535	1.000	0.423	1.000	0.500
4-methyldecane	0.678	1.406	0.441	2.219	0.275	1.000	0.500
4-methylpyrimidine	0.877	4.774	0.178	1.000	0.423	6.660	0.131
4-pyridinamine	0.974	25.105	0.039	1.000	0.423	37.157	0.026†
5-methylindane	0.600	1.000	0.535	1.000	0.423	1.000	0.500
Acetaldehyde	0.415	0.472	0.733	0.322	0.628	0.548	0.646
Acetamide	0.694	1.509	0.422	1.000	0.423	1.764	0.362
Acetic acid	0.960	15.92	0.060	2.216	0.275	22.773	0.042‡
Acetone	0.474	0.600	0.674	0.027	0.885	0.887	0.530
Acetophenone	0.845	3.629	0.224	1.000	0.423	4.943	0.168
Aromadendrene	0.904	6.245	0.141	0.953	0.432	8.891	0.101
Benzaldehyde	0.937	9.852	0.094	1.034	0.416	14.261	0.066
Benzonitrile	0.889	5.319	0.162	1.000	0.423	7.479	0.118
Benzophenone	0.994	103.841	0.010	1.000	0.423	155.261	0.006†
Benzyl acetate	0.797	2.624	0.288	1.000	0.423	3.436	0.225

	R^2	F-Statistic	p-value	Packaging F-Statistic	Packaging p-value	Ext. Time F-Statistic	Ext. Time p-value
Benzyl Alcohol	0.977	28.582	0.034	0.235	0.676	42.755	0.023†
Benzyl formate	0.744	1.937	0.358	1.000	0.423	2.406	0.294
Benzyl nitrile	0.976	26.564	0.036	1.000	0.423	39.345	0.025†
Betahistine	0.600	1.000	0.535	1.000	0.423	1.000	0.500
Butane	0.376	0.401	0.770	0.230	0.679	0.487	0.673
Butyl formate	0.687	1.466	0.430	2.399	0.262	1.000	0.500
Camphene	0.988	55.008	0.018	2.939	0.229	81.043	0.012†
Caryophyllene oxide	0.949	12.413	0.075	0.934	0.436	18.152	0.052
cis-2-pinanol	0.986	45.961	0.021	1.000	0.423	68.441	0.014†
Citronellolformate	0.971	22.408	0.043	1.000	0.423	33.111	0.029†
Decanal	0.772	2.256	0.322	1.000	0.423	2.885	0.257
Diacetone alcohol	0.730	1.804	0.376	3.413	0.206	1.000	0.500
Diethyl Phthalate	0.764	2.161	0.332	1.000	0.423	2.742	0.267
Dimethylbenzylcarbinyl acetate	0.939	10.246	0.090	1.000	0.423	14.869	0.063
Dimethylpyrazine	0.989	60.462	0.016	1.000	0.423	90.193	0.011†
Dimethylsulfide	0.702	1.567	0.412	2.700	0.242	1.000	0.500
Dimethylsulfone	0.631	1.140	0.499	1.421	0.356	1.000	0.500
DL-carvone	0.851	3.814	0.215	1.000	0.423	5.221	0.161
Dodecane	0.730	1.804	0.376	1.000	0.423	2.206	0.312
Dyclocaine	0.731	1.813	0.375	3.439	0.205	1.000	0.500
Ethanol	0.571	0.889	0.568	0.052	0.841	1.308	0.433
Ethylene oxide	0.737	1.872	0.367	1.183	0.390	2.217	0.311
Ethylenediamine	0.685	1.449	0.433	0.017	0.909	2.165	0.316
Ethylenimine	0.690	1.484	0.427	2.451	0.258	1.000	0.500
Fenchyl alcohol	0.824	3.122	0.252	1.000	0.423	4.183	0.193
Formic acid	0.600	1.000	0.535	1.000	0.423	1.000	0.500
Furfural	0.996	163.606	0.006	1.000	0.423	244.909	0.004†
Heptanal	0.955	14.188	0.067	1.000	0.423	20.782	0.046◊
Hexanal	0.981	35.257	0.028	1.630	0.330	52.070	0.019†
Hydrazine	0.956	14.586	0.065	15.147	0.060	14.306	0.065
Isoamyl alcohol	0.600	1.000	0.535	1.000	0.423	1.000	0.500
Isobornyl thiocyanoacetate	0.600	1.000	0.535	1.000	0.423	1.000	0.500
Isobutane	0.706	1.604	0.406	0.194	0.702	2.309	0.302
Isobutyraldehyde	0.595	0.981	0.541	0.975	0.427	0.984	0.504
Isocyanatomethane	0.750	1.998	0.351	3.994	0.184	1.000	0.500
Isoprene	0.789	2.499	0.299	2.204	0.276	2.647	0.274
Limonene	0.992	78.567	0.013	3.839	0.189	115.931	0.009†
Linalool	0.909	6.654	0.133	1.066	0.410	9.448	0.096
m-cymene	0.828	3.200	0.247	2.580	0.249	3.510	0.222
Methacrolein	0.954	13.700	0.069	0.024	0.892	20.537	0.046‡
Methyl acetylsalicylate	0.781	2.382	0.309	1.000	0.423	3.072	0.246
Methyl anthranilate	0.958	15.381	0.062	1.000	0.423	22.572	0.042◊
Methyl benzoate	0.970	21.864	0.044	1.000	0.423	32.296	0.030†
Methyl heptadienone	0.713	1.653	0.398	1.000	0.423	1.980	0.336
Methyl mercaptan	0.946	11.730	0.080	1.000	0.423	17.095	0.055
Methyl salicylate	0.992	81.765	0.012	1.000	0.423	122.148	0.008†
Methylene chloride	0.856	3.959	0.208	4.542	0.167	3.668	0.214
Methylisohexenyl ketone	0.986	48.075	0.020	1.466	0.350	71.380	0.014†
m-tert-butylphenol	0.675	1.384	0.445	2.153	0.280	1.000	0.500
Myrcene	0.959	15.512	0.061	1.308	0.371	22.614	0.042‡
Nerolidol	0.991	74.032	0.013	1.000	0.423	110.548	0.009†
Nonane	0.647	1.222	0.480	1.000	0.423	1.332	0.429
Octanal	0.981	33.568	0.029	1.000	0.423	49.852	0.020†

	R^2	F-Statistic	p-value	Packaging F-Statistic	Packaging p-value	Ext. Time F-Statistic	Ext. Time p-value
Pentanal	0.702	1.570	0.412	1.000	0.423	1.854	0.350
Phenol	0.676	1.389	0.445	1.000	0.423	1.583	0.387
Phenylethyl alcohol	0.999	993.769	0.001	1.000	0.423	1490.154	0.001†
Piperidine	0.600	1.000	0.535	1.000	0.423	1.000	0.500
Propanal	0.871	4.514	0.187	3.290	0.211	5.126	0.163
p-tert-butylphenol	0.600	1.000	0.535	1.000	0.423	1.000	0.500
Sabinene	0.309	0.297	0.829	0.097	0.785	0.398	0.715
Salicyladehyde	0.902	6.126	0.144	1.000	0.423	8.689	0.103
Styrene	0.870	4.465	0.188	1.000	0.423	6.197	0.139
tert-butanol	0.672	1.367	0.449	1.082	0.408	1.510	0.398
tert-butyl-benzene	0.998	280.293	0.004	1.000	0.423	419.939	0.002†
Tridecane	0.759	2.102	0.338	1.000	0.423	2.653	0.274
Tyramine	0.833	3.319	0.240	0.680	0.496	4.638	0.177
Undecane	0.748	1.984	0.352	1.000	0.423	2.476	0.288
Valencene	0.883	5.022	0.171	0.181	0.712	7.442	0.118
Verbenone	0.668	1.341	0.454	2.092	0.285	0.966	0.509
α-bisabolol	0.998	288.108	0.003	0.832	0.458	431.746	0.002†
α-cedrene	0.564	0.862	0.576	0.827	0.459	0.880	0.532
α-cubebene	0.952	13.256	0.071	1.000	0.423	19.384	0.049◊
α-guaiene	0.939	10.314	0.090	3.260	0.213	13.841	0.067
α-gurjunene	0.636	1.163	0.493	1.047	0.414	1.222	0.450
α-humulene	0.813	2.896	0.267	0.037	0.865	4.326	0.188
α-ionol	0.981	33.639	0.029	1.000	0.423	49.958	0.020†
α-longipinene	0.984	40.155	0.024	1.000	0.423	59.733	0.016†
α-phellandrene	0.535	0.767	0.609	0.564	0.531	0.869	0.535
α-pinene	0.965	18.134	0.053	1.343	0.366	26.529	0.036‡
α-terpinene	0.923	7.960	0.114	1.000	0.423	11.440	0.080
α-terpineol	0.872	4.560	0.185	0.935	0.435	6.372	0.136
β-caryophyllene	0.995	133.751	0.007	7.561	0.111	196.847	0.005†
β-cedrene	0.992	84.259	0.012	1.000	0.423	125.888	0.008†
β-pinene	1.000	2781.634	0.000	1.000	0.423	4171.951	0.000†
β-selinene	0.971	22.028	0.044	0.041	0.858	33.021	0.029†
γ-gurjunene	0.985	44.322	0.022	0.143	0.742	66.412	0.015†
γ-terpinene	0.310	0.300	0.827	0.100	0.782	0.400	0.714
δ-3-carene	0.995	144.384	0.007	1.000	0.423	216.076	0.005†

(†, 19 total) indicates significant difference in surrogate concentration of VOC at 68 h sampling time from the other two time points Tukey HSD. (‡, 5 total) indicates significant difference in surrogate concentration of the VOC between 5 min and 68 h only, (◊, 5 total) indicates no significance between extraction times after the pairwise comparison test. Statistical analysis software: XLStat V 2014.5.01 (New York, NY, USA)
