# Supplementary material for: Characterizing the Smell of Marijuana by Odor Impact of Volatile Compounds: An Application of Simultaneous Chemical and Sensory Analysis
Source: PLoS One. 2015 Dec 10;10(12):e0144160. doi: 10.1371/journal.pone.0144160 (PMC4684335; doi:10.1371/journal.pone.0144160)
Supplement: S5 Table — (PDF) [file pone.0144160.s010.pdf]

Table S5. Wilcoxon signed rank test of paired samples.

**Plastic Bag (5 min)**	
V	30
Expected value	27.500
Variance (V)	96.250
p-value (Two-tailed)	0.838
alpha	0.05

**Loose (5 min)**	
V	15
Expected value	22.500
Variance (V)	71.250
p-value (Two-tailed)	0.407
alpha	0.05

**Plastic Bag (1 h)**	
V	22
Expected value	52.500
Variance (V)	253.750
p-value (Two-tailed)	0.060
alpha	0.05

**Loose (1 h)**	
V	71
Expected value	162.500
Variance (V)	1381.250
p-value (Two-tailed)	0.014
alpha	0.05

**Plastic Bag (68 h)**	
V	245
Expected value	663.000
Variance (V)	11381.500
p-value (Two-tailed)	< 0.0001
alpha	0.05

**Loose (68 h)**	
V	143
Expected value	540.500
Variance (V)	8377.750
p-value (Two-tailed)	< 0.0001
alpha	0.05

The hypothesized difference between volatile compound abundance and odor activity value was assumed to be zero, with a 5% significance level. Test interpretation: H_0: The two samples follow the same distribution. H_a: The distributions of the two samples are different. If the computed p-value is lower than the significance level alpha=0.05, one should reject the null hypothesis H_0, and accept the alternative hypothesis H_a. Statistical analysis software: XLStat V 2014.5.01 (New York, NY, USA)
