# Supplementary material for: Characterizing the Smell of Marijuana by Odor Impact of Volatile Compounds: An Application of Simultaneous Chemical and Sensory Analysis
Source: PLoS One. 2015 Dec 10;10(12):e0144160. doi: 10.1371/journal.pone.0144160 (PMC4684335; doi:10.1371/journal.pone.0144160)
Supplement: S6 Table — (PDF) [file pone.0144160.s011.pdf]

Table S6. Hierarchy of volatile compounds with published ODT, emitted from marijuana, through packaging over 68 h.

	Plastic Bag 5 min		Plastic Bag 1 h		Plastic Bag 68 h		Loose 5 min		Loose 1 h		Loose 68 h		Duffel Bag 68 h	
Compound	[Conc]	OAV	[Conc]	OAV	[Conc]	OAV	[Conc]	OAV	[Conc]	OAV	[Conc]	OAV	[Conc]	OAV
Hydrazine	1	1			1	3	1	1	1	1	2	2	3	1
Salicyladehyde					2	28					3	28	23	39
Acetaldehyde					3	10			2	5				
Methyl benzoate					4	15					4	17	40	32
Propanal					5	26			5	16	8	23		
2-ethoxyethanol					6	7					13	8		
Phenylethyl alcohol					7	30					9	26	27	35
2-butoxyethanol					8	11					19	19		
Acetamide					9	1					28	3		
1-butanol					10	9					6	7	4	5
Styrene					11	19					17	20		
Methyl anthranilate					12	45					11	40	13	47
DL-carvone					13	31					21	31		
Pentanal					14	37					1	21		
Methylene chloride	5	2	5	2	15	4			9	2				
Benzyl acetate					16	23					26	24		
α-pinene	6	8	8	5	17	13	3	4	17	14	23	16	49	25
Methyl salicylate					18	32					15	25	14	23
1-hexanol					19	33					24	29	12	22
Isoamyl alcohol					20	35								
Octanal					21	47					14	41		
Furfural					22	14					22	13		
Piperidine					23	21							28	17
Acetophenone					24	24					10	14	17	14
2-heptanone					25	27					25	22	19	18
Phenol					26	29					39	33	24	21
2-chloroacetophenone	2	9	11	13	27	39	2	7					51	52
α-terpineol			2	9	28	38			4	13	31	34	48	42

Compound	Plastic Bag 5 min		Plastic Bag 1 h		Plastic Bag 68 h		Loose 5 min		Loose 1 h		Loose 68 h		Duffel Bag 68 h	
	[Conc]	OAV	[Conc]	OAV	[Conc]	OAV	[Conc]	OAV	[Conc]	OAV	[Conc]	OAV	[Conc]	OAV
Hexanal					29	44			6	18	30	38	5	20
Decanal					30	51					20	43	39	55
Tridecane					31	16					18	6	7	3
Nonane					32	20					7	5		
Undecane					33	22					16	10		
Formic acid					34	5							36	4
2-ethylhexanol			4	6	35	36			15	17	34	30		
Ethanol	7	5			36	8	7	3	14	4	27	4	42	6
Methylisohexenyl ketone					37	41			7	15	35	35		
Ethylene oxide	9	3			38	2					32	1		
Heptanal					39	48					37	44		
δ-3-carene					40	17					36	18		
Limonene	4	7	9	8	41	34			12	12	33	27	56	45
Isobutane			14	7	42	12	9	5	24	9	40	15	54	16
Linalool			7	10	43	43			16	20	41	39	37	37
Dodecane					44	25					29	12	43	15
α-humulene			13	14	45	42	4	6	22	21	43	37		
Acetic acid					46	40			19	19	44	36	53	41
Butane					47	6			23	3			46	2
β-caryophyllene					48	46	8	9	21	22	42	42		
Acetone	8	6	10	4	49	18	6	2	25	10	38	11	47	9
Myrcene			6	12	50	50			18	25	45	45		
Benzaldehyde			12	11	51	49	5	8	20	23	46	46	52	46
Dimethylsulfide									10	24	5	32		
Diacetone alcohol									8	7	12	9		
Piperonal													1	19
Acrolein													2	7
Nerol													6	51
Thymol													8	27
Carvacrol													9	29

Compound	Plastic Bag 5 min		Plastic Bag 1 h		Plastic Bag 68 h		Loose 5 min		Loose 1 h		Loose 68 h		Duffel Bag 68 h	
	[Conc]	OAV	[Conc]	OAV	[Conc]	OAV	[Conc]	OAV	[Conc]	OAV	[Conc]	OAV	[Conc]	OAV
p-xylene													10	10
o-guaiacol													11	49
o-methylacetophenone													15	38
Eugenol													16	34
Cumene													18	28
p-cymene													20	44
1,2,3,4-tetramethylbenzene													21	30
p-methylacetophenone													22	40
o-xylene													25	12
Durene													26	31
Methyl heptanoate													29	26
o-cymene													30	54
Anethole													31	43
Nitrobenzene													32	33
Toluene													33	13
Ethylacetate													34	11
2-butanone													35	8
Linalyl acetate													38	48
1-undecanol													41	36
3-pentanol									11	8			44	24
Propylamine													45	50
Nonanal													50	56
Isobutyraldehyde	10	10							3	11			55	53
2-nitropropane	3	4	1	1										
(+)-4-Carene			3	3					13	6				

Numbers in the table are the assigned ranking of the compound in terms of surrogate concentration ([Conc], as expressed by MS response in peak area counts) or odor impact (OAV) as calculated by Eq. 1. A rank of 1 indicates low surrogate concentration (e.g., Hydrazine from 5 min, plastic bag) or low odor impact (e.g., 2-nitropropane from 1 h, plastic bag). A rank of 56 indicates high surrogate concentration (e.g., Limonene from 68 h, duffel bag) or high odor impact (e.g., Nonanal from 68 h, duffel bag).
