# Supplementary material for: Characterizing the Smell of Marijuana by Odor Impact of Volatile Compounds: An Application of Simultaneous Chemical and Sensory Analysis
Source: PLoS One. 2015 Dec 10;10(12):e0144160. doi: 10.1371/journal.pone.0144160 (PMC4684335; doi:10.1371/journal.pone.0144160)
Supplement: S7 Table — (PDF) [file pone.0144160.s012.pdf]

Table S7. Correlation coefficients between surrogate concentration and odor impact of volatile compounds emitted from marijuana.

		R^2 (Plastic bag)			R^2 (Loose)			R^2 (Duffel bag)
X variable	Y variable	5 min	1 h	68 h	5 min	1 h	68 h	68 h
Rank [Conc]	Rank OAV	0.235	0.4211	0.1118	0.2284	0.1976	0.1693	0.1047
Rank [Conc]	[OAV]	0.2717	0.4123	0.1444	0.1001	0.172	0.1772	0.1001
[Conc]	Rank OAV	0.1062	0.3804	0.1397	0.223	0.1182	0.2232	0.1043
[Conc]	[OAV]	0.5888	0.2061	0.2806	0.1333	0.1183	0.638	0.0981

Scatter plots were generated for all combinations of rank of OAV and rank of surrogate concentration (Figure S6), actual surrogate concentration (peak area counts of mass detector, assuming equal response for all compounds) and calculated OAV (Equation 1), and correlation coefficients of the best fit line are given. The highest correlation was between surrogate concentration and calculated OAV (R^2 = 0.638) of volatiles emitted from loose, dry marijuana over 68 h at room temperature; the lowest correlation was between was between surrogate concentration and calculated OAV (R^2 = 0.0981) of volatiles emitted from fresh marijuana through a duffel bag over 68 h at room temperature.
