# Supplementary material for: Characterizing the Smell of Marijuana by Odor Impact of Volatile Compounds: An Application of Simultaneous Chemical and Sensory Analysis
Source: PLoS One. 2015 Dec 10;10(12):e0144160. doi: 10.1371/journal.pone.0144160 (PMC4684335; doi:10.1371/journal.pone.0144160)
Supplement: S8 Table — (PDF) [file pone.0144160.s013.pdf]

Table S8. Identification of VOC emitted though cloth duffel bag in headspace of marijuana sample, and captured by SPME over 68 h.

							Published ODT (ppm)			Published Descriptors			
Compound #	Aromagram #	ABC box	TIC RT (min)	AG RT (min)	Compound	CAS	LRI & Odour Database [1]	Devos, et al. [2]	Odor Descriptor	Flavornet [3]	TGSC [4]	Surrogate Conc. (PAC)	OAV
1		B	1.14		Butyl formate	592-84-7					Fruity	4.89E+04	
2		B	1.24		Isobutane	75-28-5		1.00E+01				6.00E+06	6.00E+05
3		B	1.24#		Isobutyraldehyde	78-84-2		4.07E-02		Pungent, Malt, Green	Spicy	6.00E+06	1.47E+08
4		B	1.40		2-methylpentane	107-83-5						3.95E+05	
5		B	1.40		4-methyldecane	2847-72-5						3.21E+05	
6		B	1.40		3,4,5-trimethyl-1-hexene	56728-10-0						3.21E+05	
7		B	1.45		2,2,5-trimethylhexane	3522-94-9						5.69E+04	
8		B	1.45		Isocyanatomethane	624-83-9						4.54E+04	
9		B	1.46		3-methylpentane	96-14-0						3.95E+04	
10		B	1.46		2-methylaziridine	75-55-8						8.10E+04	
11		B	1.66#		Butane	106-97-8		2.04E+02				1.28E+06	6.28E+03
12		B	1.66#		Acetone	67-64-1		1.45E+01			Solvent	1.28E+06	8.87E+04
13		B	1.77#		Methyl acetate	79-20-9					Ethereal	2.50E+05	
	1	C		1.92					Plastic, Solvent				
14		B	2.33		Propylene glycol	57-55-6						7.44E+05	
15		B	2.33#		Ethylacetate	141-78-6		2.63E+00		Pineapple	Ethereal, Fruity, Sweet, Weedy, Green	5.55E+05	2.11E+05
16		B	2.34#		Ethanol	64-17-5		2.88E+01		Sweet	Alcoholic	1.08E+06	3.74E+04
17		B	2.34		methylhydrazine	60-34-4						7.32E+05	
18		B	2.34#		Formic acid	64-18-6		2.82E+01			Acetic	7.13E+05	2.53E+04
	2	C		3.09					Onion, Garlic				
	3	C		3.31					Moldy				

Compound #	Aromagram #	ABC box	TIC RT (min)	AG RT (min)	Compound	CAS	Published ODT (ppm)			Published Descriptors		Surrogate Conc. (PAC)	OAV
							LRI & Odour Database [1]	Devos, et al. [2]	Odor Descriptor	Flavornet [3]	TGSC [4]		
19		B	3.42		3-methyl-2-cyclopenten-1-one	2758-18-1					Fruity	5.29E+04	
20		B	3.92		Hydrazine	302-01-2		3.00E+00				1.60E+04	5.34E+03
21		B	3.93		Propylamine	107-10-8		1.10E-02			Ammoniacal	1.18E+06	1.08E+08
22		B	3.93		3-pentanol	584-02-1		4.68E-01		Fruit	Herbal	1.18E+06	2.53E+06
23		B	3.93#		1,1-dimethyl-hydrazine	57-14-7						4.11E+05	
24	4	A	3.93	3.96	Ethylenediamine	107-15-3			Sweet, Solvent			3.89E+05	
25		B	5.05#		Toluene	108-88-3		1.55E+00		Paint	Sweet	4.51E+05	2.91E+05
26		B	5.06#		Phenylethyl alcohol	60-12-8		1.70E-02		Honey, Spice, Rose, Lilac	Floral	2.24E+05	1.32E+07
	5	C		5.32					Cardboard				
	6	C		5.50					Sweet, Fruity				
27	7	A	5.99#	6.00	Hexanal	66-25-1	4.00E-03	1.38E-02	Grassy, Aldehydic	Grass, Tallow, Fat	Green	2.18E+04	1.58E+06
	8	C		6.46					Sweet, Fruity				
	9	C		6.59					Onion, Skunky				
	10	C		6.92					Resiny, **Characteristic**, Onion, Skunky				
	11	C		7.74					Smoky, Resiny, Potato				
	12	C		8.36					Resiny, Potato, **Characteristic**				

Compound #	Aromagram #	ABC box	TIC RT (min)	AG RT (min)	Compound	CAS	Published ODT (ppm) LRI & Odour Database [1]	Devos, et al. [2]	Odor Descriptor	Published Descriptors Flavornet [3]	TGSC [4]	Surrogate Conc. (PAC)	OAV
28		B	8.42		2-methyl-2-propanamine	75-64-9					0	1.64E+05	
29		B	8.42		Hordenine	539-15-1					0	1.50E+05	
30		B	8.42#		2-heptanone	110-43-0	1.40E-01	1.41E-01		Soap	Cheesy	1.29E+05	9.14E+05
31		B	8.98		Hexanoic acid, methyl ester	106-70-7				Fruit, Fresh, Sweet	Fruity	3.10E+05	
32		B	9.27		Sabinene	3387-41-5				Pepper, Turpentine, Wood	Woody	3.89E+04	
	13	C		9.36					Medicinal, Herbaceous				
	14	C		9.64					Herbaceous, Medicinal				
33	15	B	10.02	9.93	β-pinene	18172-67-3			Aldehydic, Citrus, Mint, **Characteristic**	Pine, Resin, Turpentine	Terpenic	1.65E+08	
34	16	A	10.46	10.48	Isoprene	78-79-5			Moldy			2.38E+05	
35		B	10.53		Furfurylmethylamphetamine	13445-60-8						6.46E+05	
	17	C		10.64					Onion, Garlic, Skunky, Medicinal				
36		B	10.73#		1-butanol	71-36-3		4.90E-01		Medicine, Fruit	Fermented	1.82E+04	3.71E+04
37		B	10.73#		1-hexanol	111-27-3		4.37E-02		Resin, Flower, Green	Herbal	9.81E+04	2.25E+06
38		B	10.75#		Acrolein	107-02-8		1.74E-01			Almond, Cherry	8.29E+03	4.77E+04
39		B	10.90#		Limonene	138-86-3	1.00E-02	4.37E-01		Lemon, Orange	Citrus	3.48E+07	7.97E+07
40	18	A	10.90#	10.98	Camphene	79-92-5			Mushroom, Moldy	Camphor	Woody	3.48E+07	

Compound #	Aromagram #	ABC box	TIC RT (min)	AG RT (min)	Compound	CAS	Published ODT (ppm)			Published Descriptors		Surrogate Conc. (PAC)	OAV
							LRI & Odour Database [1]	Devos, et al. [2]	Odor Descriptor	Flavornet [3]	TGSC [4]		
41		B	11.28		(1R)-(+)-trans-isolimonene	5113-87-1						9.34E+05	
42		B	11.37		m-cymene	535-77-3						1.31E+05	
43		B	11.37#		p-cymene	99-87-6		2.14E-03		Solvent, Gasoline, Citrus	Terpenic	1.31E+05	6.12E+07
44		B	11.41		5-ethenyl-2-methylpyridine	140-76-1						3.21E+04	
45		B	11.48		Methyl valerate	624-24-8					Fruity	2.95E+05	
46		B	11.48		Methyl heptanoate	106-73-0		6.92E-02			Fruity	2.48E+05	3.59E+06
47		B	11.52		1,2,3,4-tetramethylbenzene	488-23-3		2.63E-02				1.41E+05	5.36E+06
48	19	A	11.54	11.55	α-phellandrene	99-83-2			Cardboard, Cabbage	Turpentine, Mint, Spice	Terpenic	8.89E+04	
49		B	11.56#		δ-3-carene	13466-78-9	4.00E+00			Lemon, Resin	Citrus	2.07E+06	
50		B	11.57		α-pinene	80-56-8		6.92E-01		Pine, Turpentine	Herbal	2.20E+06	3.17E+06
	20	C		11.91					Onion, Garlic, Skunky, Sulfury				
51	21	A	12.23#	12.23	Acetic acid	64-19-7		1.45E-01	Acidic, Burnt, Fatty acid	Sour	Acidic	4.18E+06	2.89E+07
52		B	12.51		(+)-4-Carene	29050-33-7	4.00E+00					5.94E+05	
53		B	12.51		γ-terpinene	99-85-4				Gasoline, Turpentine	Terpenic	5.68E+05	
54		B	12.82#		2-butanone	78-93-3		7.76E+00		Ether	Ethereal, Fruity, Camphor	6.62E+05	8.52E+04
55		B	13.04		4-methylphenethylamine	3261-62-9						2.00E+05	

							Published ODT (ppm)			Published Descriptors			
**Compound #**	**Aromagram #**	**ABC box**	**TIC RT (min)**	**AG RT (min)**	**Compound**	**CAS**	**LRI & Odour Database [1]**	**Devos, et al. [2]**	**Odor Descriptor**	**Flavornet [3]**	**TGSC [4]**	**Surrogate Conc. (PAC)**	**OAV**
56	22	A	13.04	13.04	o-methylacetophenone	577-16-2		6.61E-03	Moldy, Burnt		Floral	1.16E+05	1.76E+07
57		B	13.05		p-methylacetophenone	122-00-9		6.61E-03		Bitter, Almond	Floral	1.69E+05	2.56E+07
58		B	13.06#		o-xylene	95-47-6		8.51E-01		Geranium	Geranium	1.98E+05	2.33E+05
59		B	13.06#		p-xylene	106-42-3		4.90E-01				6.32E+04	1.29E+05
	23	C		13.37					Moldy, Burnt, Burnt food				
60	24	A	13.59#	13.63	Nonanal	124-19-6	1.00E-03	2.24E-03	Potato, Resiny, Roasted	Fat, Citrus, Green	Aldehydic	2.37E+06	1.06E+09
61		B	13.81		3,4,5-trimethylphenol	527-54-8					Phenolic	7.55E+05	
62	25	A	13.82	13.86	2-hydroxyacetophenone	118-93-4			Roasted, Potato, Resiny, Moldy		Phenolic	7.57E+04	
63		B	13.87		Isoquinoline	119-65-3					Balsamic	2.30E+04	
64		B	13.89		2-ethenyl-1,3-dimethylbenzene	2039-90-9						1.22E+06	
65		B	13.89		5-methylindane	874-35-1						1.22E+06	
66		B	14.04#		Benzaldehyde	100-52-7	3.00E-03	4.17E-02		Almond, Burnt sugar	Fruity	3.50E+06	8.40E+07
67		B	14.05		2-chloroacetophenone	532-27-4		2.57E-02				3.08E+06	1.20E+08
	26	C		14.15					Burnt, Burnt food				
68		B	14.17#		Dodecane	112-40-3		2.04E+00		Alkane	Alkane	1.14E+06	5.58E+05
69		B	14.19#		α-terpinene	99-86-5				Lemon	Woody	1.43E+06	
70		B	14.19		3-(1-methylethyl)-phenol methylcarbamate	64-00-6						5.04E+05	

Compound #	Aromagram #	ABC box	TIC RT (min)	AG RT (min)	Compound	CAS	Published ODT (ppm) LRI & Odour Database [1]	Devos, et al. [2]	Odor Descriptor	Published Descriptors Flavornet [3]	TGSC [4]	Surrogate Conc. (PAC)	OAV
71		B	14.21		2,4,6-trimethylphenol	527-60-6					Phenolic	1.08E+06	
72		B	14.21#		Terpinolene	586-62-9	2.00E-01			Pine, Plastic	Herbal	6.14E+05	
73		B	14.22		tert-butyl-benzene	98-06-6						2.17E+05	
74		B	14.23		o-cymene	527-84-4		7.94E-04				2.54E+05	3.20E+08
75		B	14.23#		Durene	95-93-2		2.63E-02		Rancid, Sweet	Rancid	1.99E+05	7.58E+06
76		B	14.23		Isodurene	527-53-7						1.85E+05	
77		B	14.44		1,2-diethylbenzene	135-01-3						6.97E+05	
78		B	14.45		1,4-diethylbenzene	105-05-5						7.80E+05	
79		B	14.46		3-ethyl-o-xylene	933-98-2						6.77E+05	
	27	C		14.78					Mushroom, Moldy				
80		B	15.11		Linalyl acetate	115-95-7		8.91E-03		Sweet, Fruit	Herbal	8.58E+05	9.62E+07
81		B	15.12		Linalool	78-70-6	6.00E-03	5.37E-02		Flower, Lavender	Floral	8.34E+05	1.55E+07
	28	C		15.28					Moldy, Burnt, Burnt food, Fatty acid				
82		B	15.39		Nerolidol	7212-44-4				Wood, Flower, Wax	Floral	4.33E+04	
83		B	15.40		Limonene dioxide	96-08-2					Mentholic	4.22E+04	
84		B	15.41		Methacrylic anhydride	760-93-0						2.27E+04	
85		B	15.41		Nerol	106-25-2		2.04E-04		Sweet	Floral	2.41E+04	1.18E+08
86		B	15.63#		o-dimethyl hydroquinone	91-16-7					Vanilla	2.28E+05	
87	29	A	15.64	15.68	4-methyl guaiacol	93-51-6			Herbaceous, Spicy, Burnt food, Fatty Acid, Burnt		Spicy	8.92E+04	
88		B	15.85		1-undecanol	112-42-5		6.76E-02		Mandarin	Waxy	9.21E+05	1.36E+07

Compound #	Aromagram #	ABC box	TIC RT (min)	AG RT (min)	Compound	CAS	Published ODT (ppm) LRI & Odour Database [1]	Devos, et al. [2]	Odor Descriptor	Published Descriptors Flavornet [3]	TGSC [4]	Surrogate Conc. (PAC)	OAV
89		B	15.85		Decanal	112-31-2	2.00E-03	8.91E-04		Soap, Orange peel, Tallow	Aldehydic	8.90E+05	9.99E+08
90		B	15.86#		1-hexadecanol	36653-82-4				Flower, Wax	Wax	8.06E+05	
91	30	A	15.90#	15.90	o-guaiacol	90-05-1		1.00E-03	Medicinal, Herbaceous	Smoke, Sweet, Medicine	Phenolic	9.69E+04	9.69E+07
92	31	A	16.20	16.18	5-octanolide	698-76-0			Soapy, Citrus, Mint	Peach	Coconut	6.27E+04	
93		B	16.23		Methyl benzoate	93-58-3		1.07E-01		Prune, Lettuce, Herb, Sweet	Phenolic	8.95E+05	8.35E+06
94		B	16.32#		Tridecane	629-50-5		2.14E+00		Alkane	Alkane	4.28E+04	2.00E+04
95		B	16.35		Propanoic acid, anhydride	123-62-6						8.37E+03	
96		B	16.48		3-ethyltoluene	620-14-4						8.56E+04	
97		B	16.48		p-ethyl toluene	622-96-8						8.56E+04	
98		B	16.48		2-ethyltoluene	611-14-3						7.88E+04	
99		B	16.50		Cumene	98-82-8		2.40E-02				1.18E+05	4.93E+06
100		B	16.50#		Acetophenone	98-86-2	6.50E-02	3.63E-01		Musty, Flower, Almond	Floral	1.18E+05	3.25E+05
101	32	A	16.50	16.50	Isobutyrophenone	611-70-1			Herbaceous, Medicinal		Green	8.91E+04	
102		B	16.63		Anethole	104-46-1		7.08E-03			Licorice	3.24E+05	4.58E+07
103		B	16.63		Estragole	140-67-0				Licorice, Anise	Anise	3.24E+05	
104		B	16.64		Cuminaldehyde	122-03-2				Acid, Sharp	Spicy	3.97E+05	
105		B	16.64		Pentamethylbenzene	700-12-9						3.97E+05	
106		B	16.65		Benzphentamine	156-08-1						2.82E+05	
107		B	16.65		3-isopropylbenzaldehyde	34246-57-6						3.50E+05	
108		B	16.80		Salicyladehyde	90-02-8		7.41E-03			Medicinal	1.72E+05	2.32E+07
109		B	17.10		Betazole	105-20-4						9.35E+04	
110		B	17.10		2-methyl-1H-imidazole	693-98-1						9.35E+04	

Compound #	Aromagram #	ABC box	TIC RT (min)	AG RT (min)	Compound	CAS	Published ODT (ppm) LRI & Odour Database [1]	Devos, et al. [2]	Odor Descriptor	Published Descriptors Flavornet [3]	TGSC [4]	Surrogate Conc. (PAC)	OAV
	33	C		17.16					Skunky, Sewer				
111		B	17.19		γ-hexalactone	695-06-7				Coumarin, Sweet	Tonka	2.53E+05	
112	34	A	17.41#	17.40	Piperidine	110-89-4		3.72E-01	Moldy, Burnt food		Animal	2.28E+05	6.13E+05
113		B	17.41		3-methylheptane	589-81-1						1.22E+05	
114		B	17.72		Isobornyl acetate	125-12-2					Balsamic	1.22E+06	
115		B	17.73#		α-terpineol	98-55-5		3.72E-02		Oil, Anise, Mint	Floral	1.47E+06	3.95E+07
116		B	17.73		Isobornyl thiocyanoacetate	115-31-1						1.03E+06	
117		B	17.76		Hexadecane	544-76-3				Alkane		1.30E+05	
118		B	17.76#		Pentadecane	629-62-9				Alkane	Waxy	1.30E+05	
	35	C		17.82					Citrus, Herbaceous				
119		B	17.84		Nitrobenzene	98-95-3		4.37E-02				3.81E+05	8.73E+06
	36	C		18.01					Moldy, Burnt food, Burnt				
120		B	18.04		p-acetanisole	100-06-1					Anisic	5.79E+04	
121		B	18.04		3-methyl-5-(1-methylethyl)-Phenol methylcarbamate	2631-37-0						5.79E+04	
122		B	18.04#		Carvacrol	499-75-2		1.12E-02			Spicy	5.79E+04	5.16E+06
123		B	18.04		Thymol	89-83-8		1.55E-02			Herbal	5.79E+04	3.74E+06
124		B	18.05		m-tert-butylphenol	585-34-2						7.57E+04	
125		B	18.13		α-methylcinnamaldehyde	101-39-3					Spicy	3.89E+05	
	37	C		18.37					Herbaceous, Medicinal				

Compound #	Aromagram #	ABC box	TIC RT (min)	AG RT (min)	Compound	CAS	Published ODT (ppm) LRI & Odour Database [1]	Devos, et al. [2]	Odor Descriptor	Published Descriptors Flavornet [3]	TGSC [4]	Surrogate Conc. (PAC)	OAV
126		B	18.47		α-cubebene	17699-14-8				Herb, Wax	Herb	3.21E+05	
127		B	18.73		p-aminotoluene	106-49-0						1.57E+03	
128		B	18.80		1-(3-methylphenyl)-ethanone	585-74-0						1.25E+05	
129		B	18.87#		Methyl salicylate	119-36-8	4.00E-02	4.37E-02		Peppermint	Minty	1.08E+05	2.48E+06
130		B	19.01		Hexanoic acid, propyl ester	626-77-7				Fruity	Fruity	2.41E+05	
131		B	19.09		(+)-sativene	3650-28-0						8.09E+05	
132		B	19.35		α-longipinene	5989-08-2						5.69E+05	
133		B	19.50		Perillaldehyde	2111-75-3				Spice	Herbal	2.10E+05	
134		B	19.51		(-)-Aristolene	6831-16-9						2.61E+05	
135		B	19.69		β-caryophyllene	87-44-5	6.40E-02			Wood, Spice	Spice	3.85E+07	
	38	C		19.83					Burnt food, Burnt, Piggy, Urinous				
136		B	19.85		α-guaiene	3691-12-1				Wood, Balsamic	Wood	1.68E+06	
137		B	19.96		α-bulnescene	3691-11-0						1.74E+06	
	39	C		20.20					Herbaceous, Smoky				
138	40	A	20.29#	20.29	β-cedrene	546-28-1			Herbaceous, Citrus			2.34E+06	
139		B	20.30		(+)-nerolidol	142-50-7					Floral	4.48E+06	
140		B	20.43		2-methyl naphthalene	91-57-6					Floral	6.13E+04	
141		B	20.54		α-humulene	6753-98-6	1.20E-01			Wood	Wood	1.23E+07	
142		B	20.68		Benzyl nitrile	140-29-4						2.36E+05	
	41	C		20.73					Herbaceous, Citrus, Resiny				
143		B	20.89		α-copaene	3856-25-5				Wood, Spice	Wood	4.93E+05	
	42	C		21.12					Moldy				

Compound #	Aromagram #	ABC box	TIC RT (min)	AG RT (min)	Compound	CAS	Published ODT (ppm) LRI & Odour Database [1]	Devos, et al. [2]	Odor Descriptor	Published Descriptors Flavornet [3]	TGSC [4]	Surrogate Conc. (PAC)	OAV
144	43	A	21.26	21.24	β-selinene	17066-67-0			Sweet, Fruity	Herb	Herb	6.06E+06	
145		B	21.27		(+)-calarene	17334-55-3						1.53E+06	
146		B	21.41		Aromadendrene	489-39-4				Wood	Wood	5.15E+06	
147		B	21.64#		Phenol	108-95-2		1.10E-01		Phenolic	Phenolic	1.95E+05	1.78E+06
148		B	21.69		Dyclocaine	586-60-7						2.91E+06	
149		B	21.70		Cedryl acetate	77-54-3					Wood	4.85E+05	
150		B	21.77		δ-cadinene	483-76-1				Thyme, Medicine, Wood	Herbal	7.00E+06	
151		B	21.77		α-gurjunene	489-40-7				Wood, Balsamic	Wood	5.25E+06	
	44	C		21.91					Herbaceous, Resiny				
152		B	22.08		Longifolene	475-20-7					Wood	1.43E+06	
	45	C		22.13					Gasoline, Solvent				
153		B	22.22		Valencene	4630-07-3				Green, Oil	Citrus	2.28E+07	
154		B	22.77		γ-gurjunene	22567-17-5					Musty	3.25E+06	
155		B	22.77		α-cadinene	24406-05-1					Wood	6.61E+06	
156	46	A	22.78	22.79	Alloaromadendrene	25246-27-9			Herbaceous, Resiny, Medicinal, **Characteristic**, Piggy, Urinous	Wood	Wood	4.05E+06	
157		B	23.14		2,6-dimethylquinoline	877-43-0						9.79E+05	
158		B	23.23		Tetrahydrozoline	84-22-0						7.06E+05	
159		B	23.98		Propofol	2078-54-8					Phenolic	1.80E+07	
160		B	23.98		Methyl isoeugenol	93-16-3				Clove, Spice	Spice	1.80E+07	
161		B	24.34		Isoeugenol	97-54-1				Flower	Spicy	3.41E+05	

Compound #	Aromagram #	ABC box	TIC RT (min)	AG RT (min)	Compound	CAS	Published ODT (ppm)		Odor Descriptor	Published Descriptors		Surrogate Conc. (PAC)	OAV
							LRI & Odour Database [1]	Devos, et al. [2]		Flavornet [3]	TGSC [4]		
162		B	24.34		Eugenyl acetate	93-28-7					Spicy	2.06E+05	
163		B	24.35		Carbofuran	1563-66-2						1.16E+05	
164		B	24.35		Eugenol	97-53-0		1.07E-02		Clove, Honey	Spicy	1.16E+05	1.08E+07
165		B	24.51		3-(3-hydroxyphenyl)-2-propenoic acid, methyl ester	3943-95-1						5.39E+04	
	47	C		24.64					Medicinal, Herbaceous, Resiny				
166		B	24.72		Citronellyl acetate	150-84-5				Rose, Dust	Floral	2.78E+05	
	48	C		25.07					Piggy, Urinous, Barnyard				
167		B	25.23		Methyl anthranilate	134-20-3		1.15E-03		Honey, Flower	Fruity	1.04E+05	9.04E+07
168		B	26.36		β-irone	79-70-9					Floral	2.15E+05	
169		B	26.36		2,4-di-tert-butylphenol	96-76-4					Phenolic	1.44E+05	
170		B	26.42		α-bisabolol	72691-24-8						1.24E+05	
	49	C		26.53					Piggy, Barnyard, Urinous				
171		B	26.96		7-methoxycoumarin	531-59-9				Balsamic, Sweet	Balsamic	3.15E+05	
172	50	A	27.62	27.63	Piperonal	120-57-0		4.79E-03	Potato, Resiny		Floral	6.10E+03	1.28E+06
173		B	27.86		Caryophyllene oxide	1139-30-6				Herb, Sweet, Spice	Woody	1.74E+07	
	51	C		27.98					Resiny, Potato, Roasted, **Characteristic**				

Compound #	Aromagram #	ABC box	TIC RT (min)	AG RT (min)	Compound	CAS	Published ODT (ppm) LRI & Odour Database [1]	Published ODT (ppm) Devos, et al. [2]	Odor Descriptor	Published Descriptors Flavornet [3]	Published Descriptors TGSC [4]	Surrogate Conc. (PAC)	OAV
	52	C		29.22					Potato, Resiny				
174		B	29.83		4-ethoxy-3-anisaldehyde	120-25-2					Vanilla	4.86E+04	
175	53	A	30.46	30.47	Dibutyl phthalate	84-74-2			Potato, Resiny		Bland	1.61E+04	
176		B	31.47		Hexestrol	84-16-2						1.11E+06	
177		B	31.49		p-tert-butylphenol	98-54-4					Leathery	5.84E+05	
178		B	31.79		2,6-diethylpyrazine	13067-27-1	6.00E-03				Nutty, Hazelnut	1.96E+05	

Compound # refers to the chronological order of compounds eluted off the analytical GC column; Aromagram # refers to the chronological aromagram peak order (Fig. 2). Odor descriptor refers to descriptions used by panelists in this study. Surrogate Conc. is MS response to each separated compound, in peak area counts (PAC) (i.e., the integrated area under the curve of each chromatographic peak), OAV=Odor Activity Value (ratio of surrogate concentration to odor detection threshold). # indicates compounds confirmed by pure standard for mass spectral and retention time. [1] Mottram R. LRI & Odour Database [Internet]. UK: University of Reading; [2006; cited 2014 August 08]. Available from: www.odour.org.uk/index.html [2] Devos M, Patte F, Rouault J, Laffort P, Van Gemert LJ, editors. Standardized Human Olfactory Thresholds. IRL Press at Oxford Press; 1990. [3] Acree TE, Arn H. Flavornet and human odor space [Internet]. Geneva, NY: Cornell University; [2004; cited 2014 August 08]. Available from: http://flavornet.org/flavornet.html [4] The Good Scents Company Information System [Internet]. Oak Creek, WI; [1994; cited 2014 August 08]. Available from: http://www.thegoodscentscompany.com/index.html# All ODTs presented in this table are human olfactory thresholds in air.
